# Carbon and Sulfur Cycling below the Chemocline in a Meromictic Lake and the Identification of a Novel Taxonomic Lineage in the FCB Superphylum, *Candidatus* Aegiribacteria

**DOI:** 10.3389/fmicb.2016.00598

**Published:** 2016-04-27

**Authors:** Trinity L. Hamilton, Roderick J. Bovee, Sarah R. Sattin, Wiebke Mohr, William P. Gilhooly, Timothy W. Lyons, Ann Pearson, Jennifer L. Macalady

**Affiliations:** ^1^Department of Biological Sciences, University of CincinnatiCincinnati, OH, USA; ^2^Department of Earth and Planetary Sciences, Harvard UniversityCambridge, MA, USA; ^3^Department of Earth Sciences, Indiana University-Purdue University IndianapolisIndianapolis, IN, USA; ^4^Department of Earth Sciences, University of CaliforniaRiverside, CA, USA; ^5^Penn State Astrobiology Research Center, Department of Geosciences, Pennsylvania State UniversityUniversity Park, TX, USA

**Keywords:** meromictic, euxinia, DSR, Clostridia, sulfate reducing bacteria, Deltaproteobacteria, sulfide, sulfate

## Abstract

Mahoney Lake in British Columbia is an extreme meromictic system with unusually high levels of sulfate and sulfide present in the water column. As is common in strongly stratified lakes, Mahoney Lake hosts a dense, sulfide-oxidizing phototrophic microbial community where light reaches the chemocline. Below this “plate,” the euxinic hypolimnion is anoxic, eutrophic, saline, and rich in sulfide, polysulfides, elemental sulfur, and other sulfur intermediates. While much is known regarding microbial communities in sunlit portions of euxinic systems, the composition and genetic potential of organisms living at aphotic depths have rarely been studied. Metagenomic sequencing of samples from the hypolimnion and the underlying sediments of Mahoney Lake indicate that multiple taxa contribute to sulfate reduction below the chemocline and that the hypolimnion and sediments each support distinct populations of sulfate reducing bacteria (SRB) that differ from the SRB populations observed in the chemocline. After assembling and binning the metagenomic datasets, we recovered near-complete genomes of dominant populations including two Deltaproteobacteria. One of the deltaproteobacterial genomes encoded a 16S rRNA sequence that was most closely related to the sulfur-disproportionating genus *Dissulfuribacter* and the other encoded a 16S rRNA sequence that was most closely related to the fatty acid- and aromatic acid-degrading genus *Syntrophus*. We also recovered two near-complete genomes of Firmicutes species. Analysis of concatenated ribosomal protein trees suggests these genomes are most closely related to extremely alkaliphilic genera *Alkaliphilus* and *Dethiobacter*. Our metagenomic data indicate that these Firmicutes contribute to carbon cycling below the chemocline. Lastly, we recovered a nearly complete genome from the sediment metagenome which represents a new genus within the FCB (Fibrobacteres, Chlorobi, Bacteroidetes) superphylum. Consistent with the geochemical data, we found little or no evidence for organisms capable of sulfide oxidation in the aphotic zone below the chemocline. Instead, comparison of functional genes below the chemocline are consistent with recovery of multiple populations capable of reducing oxidized sulfur. Our data support previous observations that at least some of the sulfide necessary to support the dense population of phototrophs in the chemocline is supplied from sulfate reduction in the hypolimnion and sediments. These studies provide key insights regarding the taxonomic and functional diversity within a euxinic environment and highlight the complexity of biogeochemical carbon and sulfur cycling necessary to maintain euxinia.

## Introduction

Opposing gradients of light and sulfide support phototrophic sulfur bacteria where those gradients meet. These bacteria contribute to oxidative sulfur cycling and primary productivity through carbon fixation. Mahoney Lake (ML), a small meromictic lake in British Columbia, Canada, supports a remarkably dense 10–20-cm floating “plate” of anoxygenic phototrophs in the chemocline at a depth of ~7 m, below which no light penetrates. The lake characteristics represent an extreme endmember of euxinia: a high concentration of sulfate (400–500 mM) is supplied by local surface waters draining alkaline lavas enriched in Mg^2+^, Ca^2+^, Na^+^, and ${\text{SO}}_{4}^{2-}$, and ${\text{CO}}_{3}^{2-}$, and the hypolimnion of the lake contains one of the highest levels of sulfide (30–35 mM) observed in a natural system (Northcote and Hall, 1983). The epilimnion is oxic and oligotrophic whereas the hypolimnion is eutrophic (Hall and Northcote, 1986).

At times throughout Earth's history the deep oceans have been oxygen depleted. During several of these events, regional accumulation of sulfide created areas of the ocean that were euxinic. These episodes accompanied significant events such as Phanerozoic biotic crises (e.g., Pancost et al., 2004; Grice et al., 2005) and perhaps were common in the Mesoproterozoic (Canfield, 1998; Brocks et al., 2005; Reinhard et al., 2013). Today, examples of euxinia are rare but permanently sulfidic water is observed in silled basins, meromictic lakes, coastal upwelling zones, and fjords. Of these systems, meromictic lakes are of particular interest due to their shallow phototrophic chemocline communities, extreme geochemical gradients, and laminated sedimentary records under conditions of rapid sedimentation that can preserve biosignatures (Meyer and Kump, 2008). ML is a model system as an extreme endmember of high sulfide concentration and permanent redox stability compared to other meromictic systems. Lake Cadagno, a small meromictic lake in the southern Swiss Alps, for instance is a more dilute system and experiences greater seasonal variation (Bosshard et al., 2000; Del Don et al., 2001; Decristophiris et al., 2009; Gregersen et al., 2009). The high concentration of sulfide in the water column of ML requires overloading of organic matter to support high rates of sulfate reduction. In ML, this process is driven by input of allochthonous carbon from the surrounding catchment as well as autochthonous production. The meromictic redox stability of the ML water column (permanently, rather than seasonally, stratified) results from the high salinity and high sulfur concentrations, which are promoted by the arid climate and surrounding geology (rainfall 40 cm y^−1^). The maintenance and stability of the euxinic system depends on the combination of these extrinsic factors which may contribute to their apparent rarity in other marine and nonmarine settings (e.g., Meyer and Kump, 2008; Johnston et al., 2009; Canfield, 2013; Leavitt et al., 2013).

Microbial assemblages appear to be unique within each horizon of meromictic lakes, consistent with permanent geochemical and physical partitioning, and these populations function to maintain biogeochemical cycling. In ML, the dense phototrophic plate limits the mixing of nutrients to the epilimnion and absorbs all visible light (Overmann et al., 1991, 1996a). The chemocline maintains a stable ecosystem in which complex cycling of carbon and sulfur occurs (Hamilton et al., 2014). The oxic surface waters support the growth of phytoplankton and aerobic heterotrophic bacteria (Overmann et al., 1996b); however, little is known regarding the microbial community below the chemocline where the sulfide concentration is the highest. Rates of sulfate reduction in the chemocline capable of supplying most of the sulfide necessary for anoxygenic photosynthesis are only observed in late summer (July–September; Overmann et al., 1996a). The rest of the year, molecular diffusion must supply a significant fraction of sulfide to support the dense phototrophic layer, assuming the layer is very active throughout the year. Sulfate concentrations in the monimolimnion were similar throughout the year while the sulfide concentrations ranged from 27 to 60 mM during the study (Overmann et al., 1996a). Collectively, these data provide few clues regarding biological activity in the monimolimnion and sediments of the lake. 16S rRNA analyses of ML indicate that each stratified layer is taxonomically distinct (Klepac-Ceraj et al., 2012). These data also suggest that microbial communities in the deep waters and sediments of Mahoney Lake make important contributions to biogeochemical cycling in the lake through sulfate reduction and fermentation.

Here we report the results of metagenomic sequencing of the ML community from 8 m, just under the phototrophic plate, and from the lake bottom sediment, (15 m) as obtained by grab core. Our data reveal the presence of anaerobic bacteria involved in remineralization of organic matter below the chemocline. Consistent with the high levels of sulfide in ML, we observed populations of sulfate reducing bacteria at 8 m and in the sediments. Genomic reconstruction resulted in two genomes of Deltaproteobacteria species, two genomes of Firmicutes species, and a genome of a species from the FCB (Fibrobacteres, Chlorobi, Bacteroidetes) superphylum which represents a new genus within this superphylum. Collectively, our data provide key insight into biogeochemical cycling in a modern, extreme analog of early Earth euxinia.

## Materials and methods

### Environmental sample collection and DNA extraction

Water from 8 m was collected using a Niskin bottle and a grab core was collected of the underlying sediments (15 m) from ML, British Columbia (49° 17′N, 119° 35′W) in July 2008. 50 mL samples of water and sediments were immediately frozen on dry ice for transport to Harvard University, where they were stored at −80°C until further processed. For extraction of genomic DNA, samples were thawed and centrifuged, and DNA was extracted from the resulting pellets using an e.Z.N.A SP Plant Maxi Kit (Omega Bio-tek, Norcross, Georgia) according to the manufacturer's instructions. The yield and quality of the extracted DNA were assessed using gel electrophoresis visualized by ethidium bromide staining and spectrophotometry using a NanoDrop ND-1000 spectrophotometer (NanoDrop Technologies, Wilmington, Delaware).

### Metagenomic sequencing, assembly, and binning

Fragmentation and library preparation were performed by the North Carolina State University Genomic Sciences Laboratory. Paired-end 150 bp Illumina (HiSeq 2500) sequencing was performed at the Harvard Center for Systems Biology. Reads were trimmed with Trimmomatic 0.20 (Lohse et al., 2012), and only sequences with at least 50 base pairs in both the forward and reverse direction were retained. Trimmed, screened, paired-end Illumina reads were assembled into contigs with IDBA-UD (ver. 1.1.1) using eight threads with default parameters (Table S1; Peng et al., 2012). Coverage was determined by aligning raw reads to contigs using BWA 0.5.9 (Li and Durbin, 2009).

The assembled contigs were annotated with an in-house annotation pipeline as described previously (Hamilton et al., 2014). Briefly, rRNAs were identified using Meta_RNA (Huang et al., 2009) and Phyloshop (Shah et al., 2010), and tRNAs were identified with tRNAScan (Lowe and Eddy, 1997). Protein-coding genes were identified using the *ab initio* gene calling tools GeneMark (v.2.6r) (Lukashin and Borodovsky, 1998), MetaGene (v. Aug08) (Noguchi et al., 2006), Prodigal (Hyatt et al., 2010), and FragGeneScan (Rho et al., 2010). Genes were associated with COGs (Clusters of Orthologous Groups of proteins) using rpsblast (Tatusov et al., 2001) and Pfam with hmmsearch (Durbin et al., 1998). Amino acid similarity searches were used for assignment of KO terms (KEGG) (Ogato et al., 2000) and EC numbers to open reading frames.

A custom Python script (available at https://github.com/bovee/Ochre) was used to calculate tetranucleotide frequency of all contigs ≥2500 bp. Corresponding reverse-complement tetranucleotides were combined as described (Dick et al., 2009). Contigs were then binned using emergent self-organizing maps (ESOM) based on tetranucleotide frequency, which resulted in clusters corresponding to taxonomically sorted tetranucleotide usage patterns (Dick et al., 2009). For binning, contigs were split into 5000-bp segments, clustered into taxonomic groups (or “genomic bins”; Voorhies et al., 2012) by tetranucleotide frequency and visualized with Databionic-ESOM (http://databionic-esom.sourceforge.net) using parameters from Dick et al. (2009). Following manual inspection for homogeneous read coverage and further curation by BLASTX/N, phylum-level taxonomic assignment was performed using Phyloshop (Shah et al., 2010) and Megan (Huson et al., 2011).

Well-defined, high coverage bins were selected for in-depth characterization and taxonomic assignment of their predicted genes. Paired reads mapping to scaffolds from each bin were reassembled using Velvet (Zerbino and Birney, 2008) or IDBA-UD (ver. 1.1.1) as previously described (Hug et al., 2013). Scaffolds of each re-assembly were annotated as described above. To estimate genome completeness, the presence of a suite of 76 genes selected from a set of single-copy marker genes that show no evidence for lateral gene transfer (Sorek et al., 2007; Wu and Eisen, 2008) was evaluated (Table S2). Genome coverage was estimated by assuming that the genome size of each phylotype was approximately the same as its closest relative (Whitaker and Banfield, 2006; Jones et al., 2012). Average nucleotide identity (ANI) of protein-coding genes between genomes was calculated using the ANIb BLAST+-based analyses within the JSpeciesWS (Richter et al., 2015).

### 16S rRNA gene reconstruction

Near full-length 16S rRNA sequences were reconstructed from Illumina sequencing reads using EMIRGE (Miller et al., 2011). EMIRGE was run for 100 iterations with default parameters designed to merge reconstructed 16S rRNA genes if candidate consensus sequences share ≥97% sequence identity in any iteration. The non-redundant SILVA SSU reference database version 111 (http://www.arb-silva.de/) was used as the starting database of curated SSU sequences. The relative abundance of each OTU was calculated statistically via the EMIRGE algorithm based on “prior probabilities” of read coverage depth (Miller et al., 2011). Sequences with an estimated abundance of < 0.01% were removed from further analyses. Potential chimeras were identified with UCHIME (Edgar et al., 2011) using Mothur (ver 1.32.1; Schloss et al., 2009) and removed from further analyses. Taxonomic assignment of the EMIRGE-reconstructed 16S rRNA sequences was performed using BLAST and ARB (Ludwig et al., 2004).

### Taxonomic assignment of genome bins

Several different marker sequences were used to robustly assign taxonomy of the genome bins including 16S rRNA gene sequences (if present in the bin) and ribosomal proteins encoded in a syntenous block (Table S3). When present, the phylogenetic position of 16S rRNA genes was used to make genus-level assignments of genomic bins. The 16S rRNA gene sequences from the genomic bins and closely related sequences identified with BLASTN searches were aligned to the SILVA reference alignment using the SINA Webaligner and merged into the SILVA version 108 database (Pruesse et al., 2007). The alignment was manually refined in ARB (Ludwig et al., 2004), and neighbor-joining analyses were performed in PAUP^*^ v. 4b10 (Swofford, 2001) using Jukes-Cantor-corrected distances and 1000 bootstrap replicates.

Single-copy ribosomal proteins were also analyzed to make genus-level assignments of genomic bins independently from the 16S rRNA gene sequences and in cases where 16S rRNA gene sequences were not present in the bins. Such analyses yield resolution comparable to that for 16S rRNA phylogenetic trees (Hug et al., 2013). A subset of single copy phylogenetic marker proteins (*n* = 18), identified by Phylo-AMPHORA (Wang and Wu, 2013; Table S3) and verified by annotation and BLAST, were used to assign phylotypes. Reference datasets were further populated with sequences mined from genome sequences in the NCBI databases and JGI IMG-M. Each protein was aligned individually using MEGA (version 6.0, Tamura et al., 2013), and evolutionary models were determined using ProTest (version 3, Darriba et al., 2012). Alignments were then concatenated and neighbor-joining and maximum likelihood phylogeny of each concatenated alignment was calculated using PhyML (Guindon and Gascuel, 2003) and the ProTest-determined evolutionary model with 1000 bootstrap replicates.

### Comparative analysis

Through the annotation pipeline describe above, genes were associated with COGs using rpsblast (Tatusov et al., 2001). The abundance of individual reads matching a particular COG were converted to a fraction representing the relative contribution of each COG count to the total number of sequences assigned to COGs for each dataset (8 m and sediment) to account for different levels of sampling across multiple datasets (Konstantinidis et al., 2009; White et al., 2009; Ferreira et al., 2014). This method has been shown to reduce annotation bias (Delmont et al., 2011). For analysis of carbon, nitrogen, and sulfur cycling pathways, marker genes were defined as previously described (Lauro et al., 2011; Llorens-Marès et al., 2015) and normalized. A full list of the genes is provided in Table S4. Hierarchical cluster analysis and heat map plots were generated with R (R Development Core Team, 2008) using the library “seriation.” The marker genes for dissimilatory sulfate reduction and sulfide oxidation (K00394, K00395, K00396) can operate in both sulfide oxidation and sulfate reduction. Therefore, they were assigned to sulfate reduction or sulfur oxidation based on the best match within KEGG.

### Nucleotide sequence accession numbers

The assembled metagenomic sequences can be accessed via IMG/M (http://img.jgi.doe.gov). Raw sequence reads of all samples were deposited at the NCBI Short Read Archive (SRA) and can be accessed under the accession numbers SRR2986055 (8-m sample) and SRR2989655 (sediments). Metagenome bin sequences—ML8_D, MLS_D ML8_F1, ML8_F2, and MLS_C—are deposited at DDBJ/EMBL/GenBank under the accession numbers SAMN04330442, SAMN04330450, SAMN04330440, SAMN04330448, and SAMN04330451, respectively.

## Results and discussion

### Community structure below the chemocline

**Figure 1:**
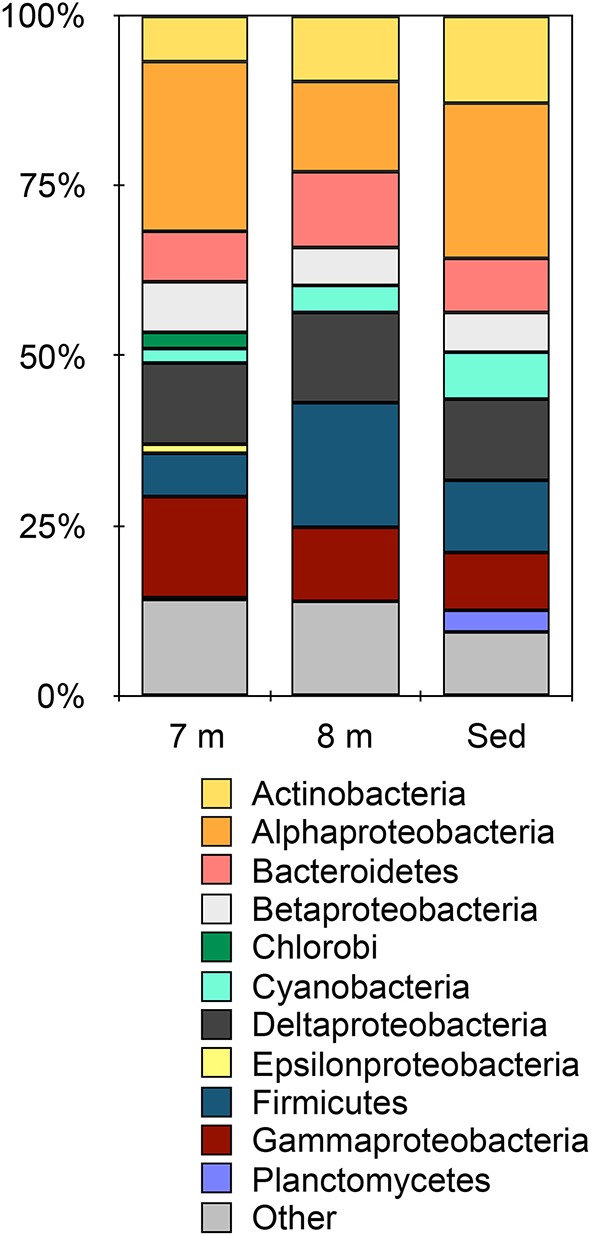
**Taxonomic affiliation of sequences in the metagenomes assigned at the phylum-level (except the Proteobacteria which are represented by class)**. Other indicates all phyla represented by < 5% of the total sequences in any metagenome. Data for 7 m are from Hamilton et al. (2014).

Metagenomic data from 8 m water depth and the sediments from Mahoney Lake resulted in 364,727 contigs containing ~496 Mbp and 593,174 contigs containing ~781 Mbp, respectively. Based on BlastX, the majority of contigs (>92%) in each metagenome were assigned to Bacteria while small numbers of sequences affiliated with Archaea, Eukaryota, and viruses were also recovered (Figure S1). Despite changes in the redox chemistry of the water column (Northcote and Hall, 1983; Overmann et al., 1991, 1996a) and the close proximity of the 8-m sample to the dense phototrophic plate at 7 m, the microbial community at 8 m is remarkably similar at the phylum level to the sediments (Figure 1). Within the Bacteria, the largest number of sequences were assigned to the Firmicutes and Alpha-, Delta,- and Gammaproteobacteria at both 8 m and in the sediments (Figure 1). This observation is consistent with the recovery of lipids common to Deltaproteobacteria and heterotrophic, Gram-positive bacteria such as Firmicutes from these samples (Bovee and Pearson, 2014). Sequences affiliated with the Alphaproteobacteria were the most abundant in the 7-m (Hamilton et al., 2014) and sediment samples, accounting for more than 24% and more than 22% of the total, respectively. Sequences affiliated with the Firmicutes were the most abundant group from 8 m, where they accounted for 18% of the total sequences. Sequences affiliated with the Cyanobacteria were present in all samples, while sequences affiliated with Planctomycetes were present only in the sediments. Our previous observations of the chemocline community (Hamilton et al., 2014) and those presented here suggest that phylum-level similarities mask the true level of microbial complexity, which imply variations in the biogeochemical functions being carried out in each environment of the lake.

**Figure 2:**
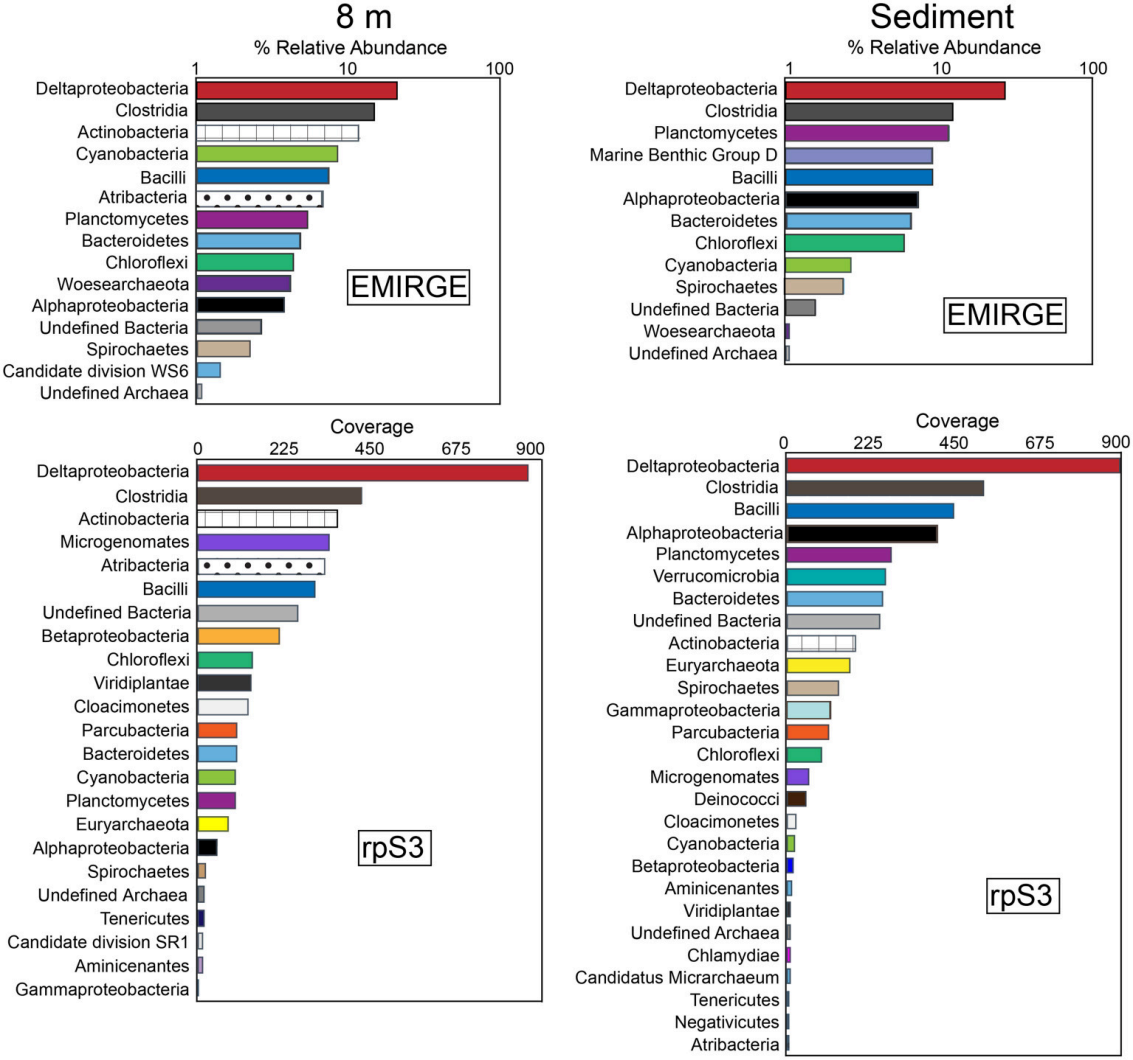
**Rank-abundance curves of EMIRGE 16S rRNA sequences and ribosomal protein S3 (rpS3) sequences from the 8 m and sediment metagenomes**. The relative abundance of 16S rRNA genes was calculated with EMIRGE (Miller et al., 2011). The rank abundance curves for rpS3 sequences are based on average depth of coverage of the contig where each rpS3 sequence was encoded. Bars represent the sum of the relative abundance of taxonomic groups (at the phylum level except for Proteobacteria and Firmicutes which are summed at the class level).

Analysis of full-length 16S rRNA gene sequences reconstructed using EMIRGE resulted in 35 unique operational taxonomic units (OTUs) from both the 8-m and the sediment samples. Rank-abundance curves of the EMIRGE-reconstructed 16S rRNA gene sequences indicate that sequences affiliated with the Deltaproteobacteria and Clostridia are the most abundant in both the 8-m and sediment sample (Figure 2). At 8 m, the relative abundance of 16S rRNA sequences affiliated with Actinobacteria, Cyanobacteria, and Bacilli was higher than in the sediments. In contrast, 16S rRNA sequences affiliated with Planctomycetes and Marine Benthic Group D, an uncultured archaeal clade (Vetriani et al., 1999), were more abundant in the sediments. The recovery of Cyanobacterial 16S rRNA sequences below the sunlit zone suggests sinking biomass from the oxygenated layer of the lake above the chemocline, with preservation of their DNA promoted by the sulfidic, anoxic environment (Coolen and Overmann, 1998). It is worth noting that these 16S rRNA sequences were most closely related to typical phototrophic Cyanobacteria as opposed to the recently discovered non-photosynthetic Melainabacteria which form a novel candidate phylum sibling to Cyanobacteria (Di Rienzi et al., 2013). No observations have been reported of Cyanobacteria thriving in sulfide-rich water below the chemocline in a permanently stratified lake.

In addition to the analysis of EMIRGE-reconstructed 16S rRNA gene sequences, we also examined ribosomal protein S3 (rpS3) sequences. rpS3 has a strong phylogenetic signal (Brown et al., 2015) and unlike the EMIRGE-reconstruction that is independent of assembly, provides a direct marker for community composition in the assembled metagenomic data. Using read coverage depth, we also constructed rank-abundance curves of rpS3 sequences from the 8-m and sediment metagenomes. The rank abundance curves for rpS3 sequences are based on average depth of coverage of the contig where each rpS3 sequence was encoded. In general, the rank abundance curves for the rpS3 sequences were in agreement with those observed for the 16S rRNA gene sequences—the highest coverage rpS3 sequences at 8 m and in the sediments were affiliated with Deltaproteobacteria and Clostridia (Figure 2). Actinobacteria and Bacilli rpS3 sequences were abundant in the 8-m sample, and Planctomycetes were abundant in the sediments. In the 8-m sample, rpS3 sequences affiliated with Microgenomates (OP11) and Atribacteria were also abundant.

Some of the highest coverage 16S rRNA and rpS3 sequences were affiliated to lineages with no cultured representatives. For instance, 16S rRNA sequences affiliated with the recently described Woesearchaeota were identified at 8 m. The Woesearchaeota are a new phylum-level lineage within the DPANN superphylum of Archaea with no cultured representatives, despite recovery of multiple genomes of this lineage from groundwater and sediment samples (Castelle et al., 2015) as well as from Sakinaw Lake (Rinke et al., 2013). Sakinaw Lake is a coastal meromictic lake in British Columbia, Canada, with a mixolimnion at 30 m and an anoxic monimolimnion with high concentrations of sodium chloride, sulfate, and sulfide (Perry and Pedersen, 1993; Vagle et al., 2010; Gies et al., 2014). Sequences affiliated with Atribacteria (OP9 and JS1) were also abundant at 8 m. Members of the Atribacteria have been detected in a variety of environments including geothermal systems, petroleum reservoirs, anaerobic digesters, and wastewater treatment facilities. Sequences affiliated to Atribacteria are also found in anaerobic, methane hydrate-bearing sediments (Inagaki et al., 2006; Carr et al., 2015), including those that are low in sulfate. Single-cell and metagenomic sequencing suggests members of the Atribacteria are not involved in sulfate reduction (Dodsworth et al., 2013; Nobu et al., 2015a) but are anaerobic sugar fermenters. Notably, Atribacteria were also recovered from Sakinaw Lake (Rinke et al., 2013; Gies et al., 2014). 16S rRNA sequences affiliated Archaea of Marine Benthic Group D (MBG-D) were abundant in the Mahoney Lake sediments. Members of MBG-D are among the most numerous Archaea in sediments underlying the Earth's oceans (Lloyd et al., 2013). rpS3 sequences affiliated with the Microgenomates (OP11) were present in high coverage at 8 m. Sequences affiliated with Microgenomates have been detected in many environmental samples (Harris et al., 2004) including in anoxic, carbon-rich environments (Briée et al., 2007), but no cultured representatives have been characterized. Metagenomic sequencing suggests a lifestyle based on fermentation (Wrighton et al., 2012).

### Potential for sulfate reduction

Sulfate reducing bacteria and archaea include members of the phyla Proteobacteria (Deltaproteobacteria), Firmicutes, Nitrospirae, Thermodesulfobacteria, Crenarchaeota, and Euryarchaeota. In general, these taxa couple the oxidation of organic matter or H_2_ to the reduction of sulfate or compounds with intermediate sulfur oxidation states to sulfide. Sulfide concentrations reach 30–35 mM in the hypolimnion of ML (Overmann et al., 1996a), exceeding the concentration thought to inhibit sulfate reduction (~16 mM; (Reis et al., 1992)). At elevated levels, sulfide is reversibly toxic. However, the H_2_S:${\text{SO}}_{4}^{2-}$ ratio in this system still is not high enough to thermodynamically inhibit sulfate reduction because of the very high levels of associated sulfate (Amend and Shock, 2001; Hamilton et al., 2014). Here, we classified rpS3 sequences affiliated with known sulfur reducing bacteria (Deltaproteobacteria and Clostridia were the only ones detected) to better understand the potential for sulfate reduction below the chemocline in ML.

**Figure 3:**
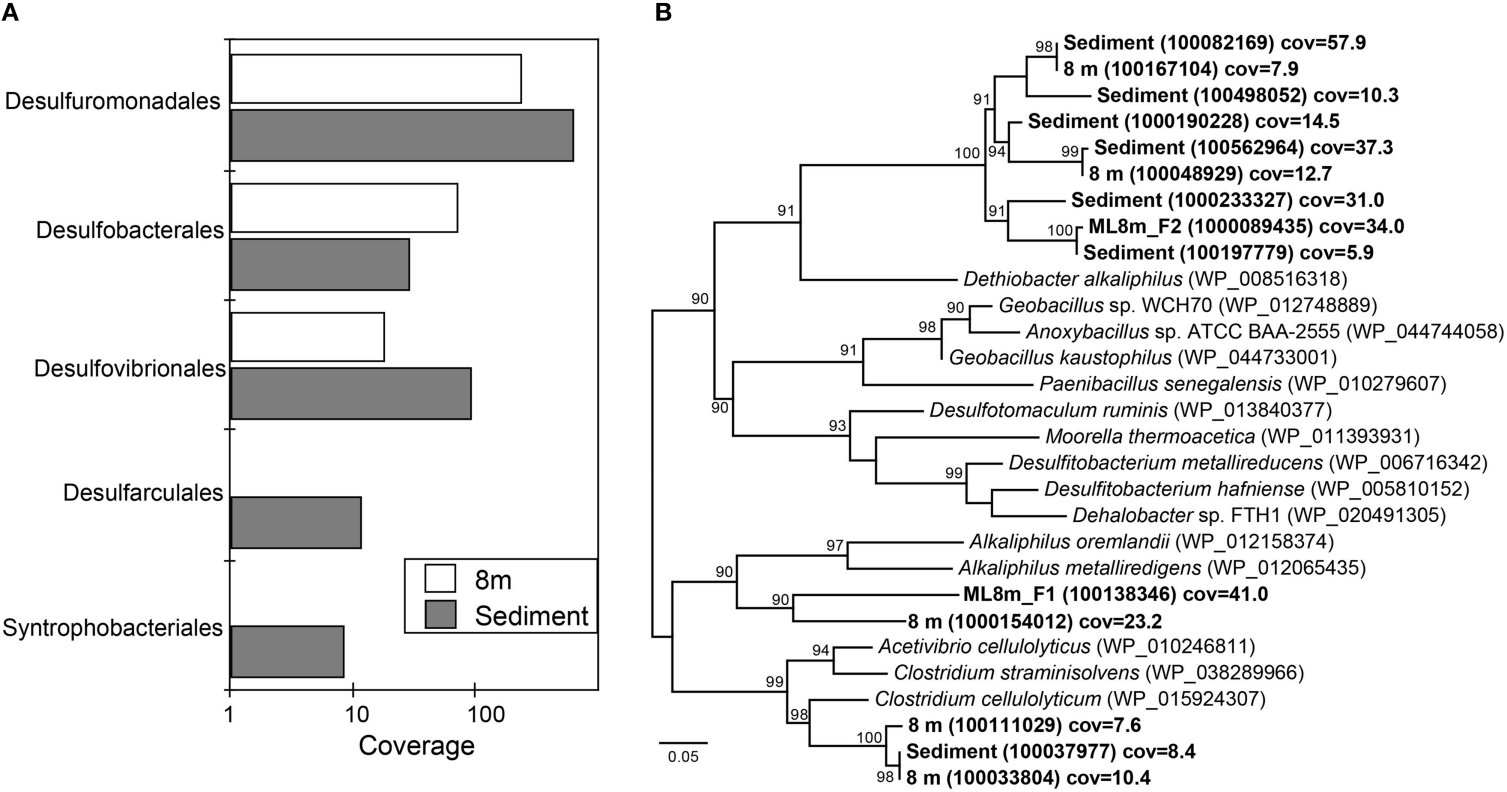
**Phylogenetic classification of ribosomal protein S3 (rpS3) sequences affiliated with Deltaproteobacteria (A) and Clostridia (B). (A)** Rank-abundance curve of Deltaproteobacterial ribosomal protein S3 (rpS3) sequences from the 8 m and sediment metagenomes classified at the order level. **(B)** Maximum likelihood phylogenetic tree of rpS3 sequences affiliated with Clostridia recovered from the 8 m and sediment metagenomes and closely related sequences. Sequences recovered in the present study are in bold. NCBI accession numbers or IMG gene ids are shown in parentheses. Bootstrap support values based on 1000 bootstrap samplings >90 are noted. Cov = average depth of coverage of the contig containing the rpS3 sequence.

Sequences affiliated with the *Desulfuromonadales* are present in high coverage at both 8 m and in the sediments based on rank abundance curves of Deltaproteobacterial rpS3 sequences (Figure 3A). The hypolimnion (8 m) and sediments are also rich in both elemental sulfur and polysulfide and most members of the *Desulfuromonadales* couple the complete oxidation of organic substrates to CO_2_ with the reduction of S^0^ or Fe(III). Sequences affiliated with the *Desulfobacterales* were present in highest coverage at 8 m (Figure 3A). Members of the *Desulfobacterales* grow best when coupling acetate oxidation to sulfate reduction although some species are capable of fixing CO_2_. Acetate was the main volatile fatty acid accumulated in ML chemocline incubations when sulfate reduction was inhibited by molybdate, suggesting it is the preferred carbon source of sulfate reducers within the chemocline (Overmann et al., 1996a). Our detection of *Desulfobacterales* at 8 m suggests that acetate is also important in the hypolimnion. *Desulfobacterales* species are common in anaerobic marine and brackish sediments (Widdel, 1987).

*Desulfovibrionales* were also relatively more common in ML sediments than in the water column (Figure 3A). While these taxa also reduce sulfate to sulfide, the Order *Desulfovibrionales* includes spp. that are capable of switching to a syntrophic lifestyle based on hydrogen consumption (Bryant et al., 1977; McInerney and Bryant, 1981; Stolyar et al., 2007). rpS3 sequences affiliated with the *Syntrophobacteriales* also were recovered from the sediments (Figure 3A). Members of the *Syntrophobacteriales* are syntrophic, usually with hydrogen- or formate-utilizing organisms, and are capable of fueling sulfate reduction, and typically degrade fatty or aromatic acids. Together, these results point to the likely importance of H_2_-based syntrophy in ML sediments. Syntrophic metabolisms are common in many anoxic environments where they are integral to carbon cycling (Morris et al., 2013). In characterized *Desulfovibrionales* species, the switch to syntrophy occurs in the absence of sulfate. In contrast to this observation, the recovery of sequences affiliated with syntrophic sulfate reducing organisms from the sulfate-rich deep ML environment suggests that syntrophic interactions mediated by Deltaproteobacteria might contribute to the anoxic carbon cycle in Mahoney Lake. Alternatively, members of the *Desulfovibrionales* and *Desulfobacterales* are also capable of fueling cell growth via disproportionation of sulfite or thiosulfate to sulfate and sulfide (Bak and Pfennig, 1987). rpS3 sequences affiliated with the *Desulfovibrionales* and *Desulfobacterales* were also recovered from the chemocline of Mahoney Lake, where disproportionation rather than (or in addition to) H_2_-based syntrophy is thought to be important (Hamilton et al., 2014).

We recovered 14 unique rpS3 sequences affiliated with Clostridia below the chemocline. The majority of these sequences were most closely related to the alkaliphilic *Dethiobacter alkaliphilus* (Figure 3B). *Dt. alkaliphilus* spp. can grow by sulfur disproportionation, and polysulfides are important intermediates during this reaction (Poser et al., 2013). Geochemical data collected *in situ* indicates zero valent sulfur, including both intracellular sulfur and polysulfides, are important intermediates in the S cycle in ML (Overmann et al., 1996a). Polysulfide concentrations in the bulk chemocline water of Mahoney Lake are around 50 μM and increase to ~400 μM at a depth of 13–14 m (Overmann et al., 1996a). Other rpS3 sequences were affiliated with *Alkaliphilus metalliredigenes, Alkaliphilus oremlandii*, and *Clostridium celluloyticum* (Figure 3B). *A. oremlandii* is a spore-forming organism that can use arsenate or thiosulfate as an electron acceptor with small organics such as acetate, pyruvate, or lactate as electron donors (Fisher et al., 2008). *A. metalliredigenes* was isolated from a high-pH borax leachate pond. It reduces metals in the presence of yeast extract at elevated salt concentrations; however use of sulfate or thiosulfate as an electron acceptor in pure culture was not observed (Ye et al., 2004). *C. celluloyticum* and other closely related *Clostridium* species degrade cellulose, xylan and polysaccharides.

### Community metabolism

**Figure 4:**
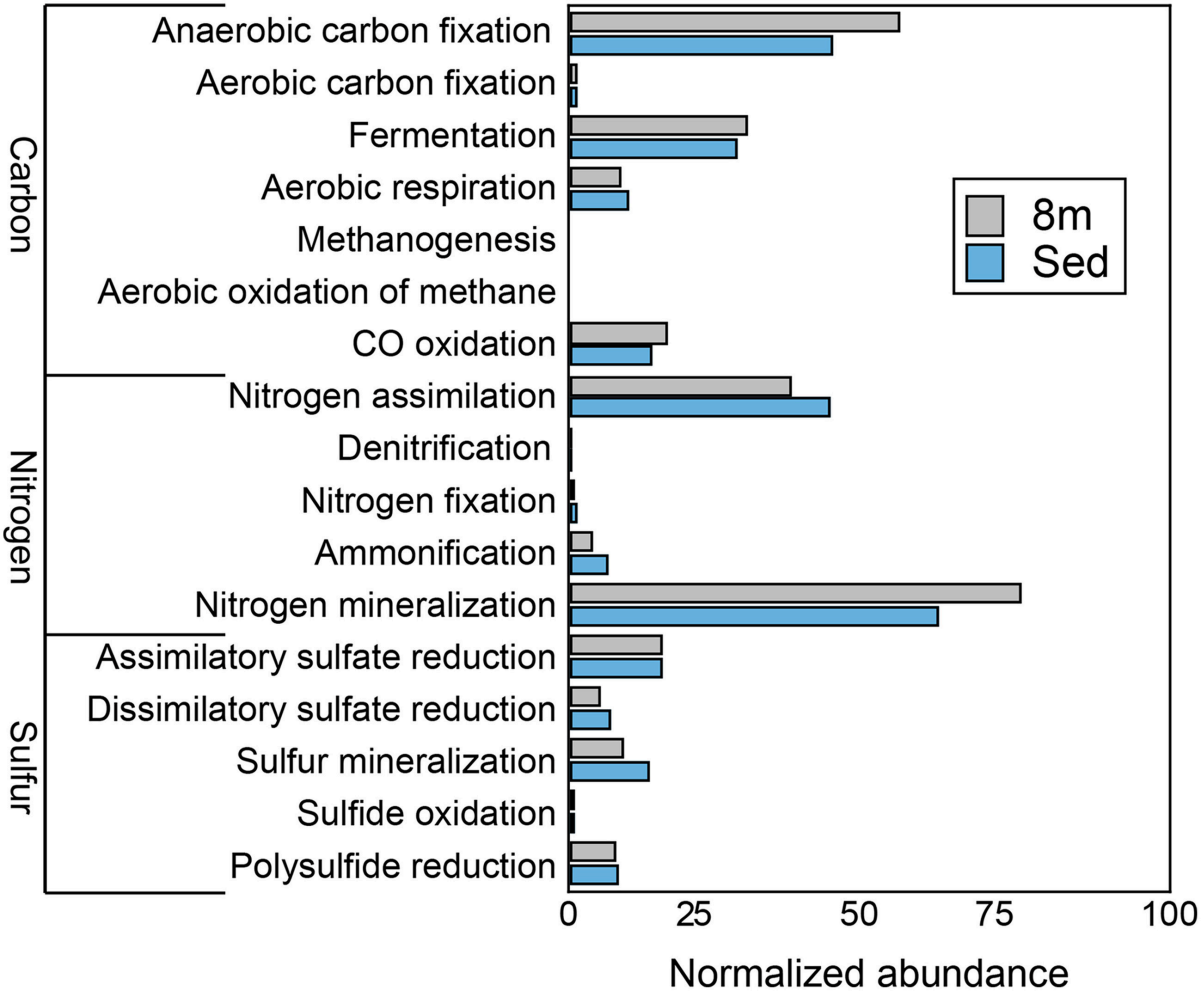
**The genetic potential for carbon, nitrogen, and sulfur cycling at 8 m and in the sediments of Mahoney Lake**. The genetic potential for each step was estimated using a combination of normalized marker gene ratios as previously described (Lauro et al., 2011). The marker genes for dissimilatory sulfate reduction and sulfide oxidation (K00394, K00395, K00396) can operate in both sulfide oxidation and sulfate reduction. Therefore, they were assigned to sulfate reduction or sulfur oxidation based on the best match within KEGG. Sed, sediments. Marker genes are provided in Table S4.

Consistent with the observation of anoxic water below the chemocline, we detected very few genes that encode components of aerobic carbon fixation or aerobic respiration (Figure 4). The genetic potential for fermentation and CO oxidation was moderately more abundant at 8 m than in the sediments. We observed no evidence for methanogenesis or the aerobic oxidation of methane at either depth. Genes for nitrogen mineralization and nitrogen assimilation were abundant at both depths. The 8-m and sediment metagenomes contain a limited diversity of functional genes for assimilatory and dissimilatory sulfate reduction. To further assess the genetic potential for sulfate reduction at 8 m and in the sediments, we identified three genes essential for sulfate reduction: *dsrAB*, the genes encoding dissimilatory sulfite reductase, and *dsrC*. The 8-m metagenome contained five copies of *dsrAB*, and the sediment metagenome contained 12 copies of *dsrAB.* We also recovered 17 copies of *dsrC*, that all encode the two strictly conserved cysteines in the C-terminal arm (Santos et al., 2015), from the 8-m metagenome and 17 from the sediments. *dsr* genes are also encoded in the genomes of non-sulfate-reducing, syntrophic bacteria and thus their presence does not confirm sulfate reduction (Imachi et al., 2006). Regardless, the closest tBlastN hits of all of the *dsrAB* and *dsrC* sequences were to dissimilatory sulfate reducing Deltaproteobacteria, suggesting they are not involved in sulfur oxidation. Genes encoding components of the Sox enzyme complex which oxidizes thiosulfate were rare, as were genes coding for sulfide quinone oxidoreductase, a protein that oxidizes sulfide to elemental sulfur (data not shown).

Organisms such as Clostridia produce H_2_ to dispose of the excess reductant generated during fermentation, while others may also produce H_2_S when polysulfide is available (Vignais et al., 2001). In general, the H_2_ evolution activity of [FeFe]-hydrogenases is 10- to 100-fold greater than [NiFe]-hydrogenases that are typically involved in H_2_ uptake (Vignais et al., 2001; Frey, 2002). Fermentative organisms can also consume H_2_ via [NiFe]-hydrogenases to produce the reduced form of nicotinamide adenine dinucleotide phosphate (NADPH) for anabolic metabolism. The 8-m metagenome contained 154 copies of genes encoding [NiFe]-hydrogenases and 163 copies of genes encoding [FeFe]-hydrogenases, while the sediment dataset contained 242 genes encoding [NiFe]-hydrogenases and 199 copies of genes encoding [FeFe]-hydrogenases. This distribution is consistent with the high abundance of organisms related to known fermenters and with the presence of other genes involved in fermentation. The 8-m metagenome contained 32 copies of the gene encoding the NifD, a structural component of the Mo-dependent nitrogenase, while 40 copies of *nifD* were recovered from the sediment metagenome. N_2_ reduction, catalyzed by the nitrogenase enzyme, results in H_2_ production concomitant with N_2_-fixation with a fraction of the electrons used for H_2_ production (Eady, 1996). In environments where fixed nitrogen is not limiting, an intriguing possibility is that nitrogen fixation is employed for H_2_ production. The metagenomes also encode numerous proteins that may be involved in the decomposition of recalcitrant plant biomass or cellulose, including glycosyl hydrolyses, glycosyl transferases, endoglucanases, and glyoxal oxidases.

We recovered 12 and 15 full-length, unique sequences of the gene encoding ribulose-1,5 bisphosphate carboxylase-oxygenase (RuBisCO), the key CO_2_-fixing enzyme of the Calvin-Benson-Bassham (CBB) cycle, from the 8-m metagenome and the sediment metagenome respectively. Of the 27 sequences, eight were identified as type I, five as type II, 10 as type III, and five as type IV (Figure S2). RuBisCO type I is most efficient under low CO_2_ concentration while Type II is less able to discriminate between O_2_ and CO_2_ and thus tends to function better under low oxygen and/or high CO_2_ concentrations. Type III RuBisCO enzymes are typically associated with Archaea; however, sequences affiliated with archaea were not abundant in either metagenome. In Archaea, the type III enzymes are involved in converting AMP, phosphate, CO_2_, and H_2_O to adenine and two molecules of 3-PGA, salvaging the adenine base of AMP (Sato et al., 2007; Wrighton et al., 2012). Type IV RuBisCO or RuBisCO-like protein is thought to be incapable of catalyzing carbon fixation because it lacks several key conserved residues necessary for carboxylase activity; instead, RuBisCO-like protein may play a role in sulfur oxidation or oxidative stress (Tabita et al., 2007).

### Genomes

Analysis of tetranucleotide frequency allowed the recovery of several near-complete genomes, including two Firmicutes species from the 8-m sample, a genome of a new member of the FCB superphylum from the sediments and two Deltaproteobacteria species—one from 8 m and one from the sediment sample.

#### Deltaproteobacteria

**Figure 5:**
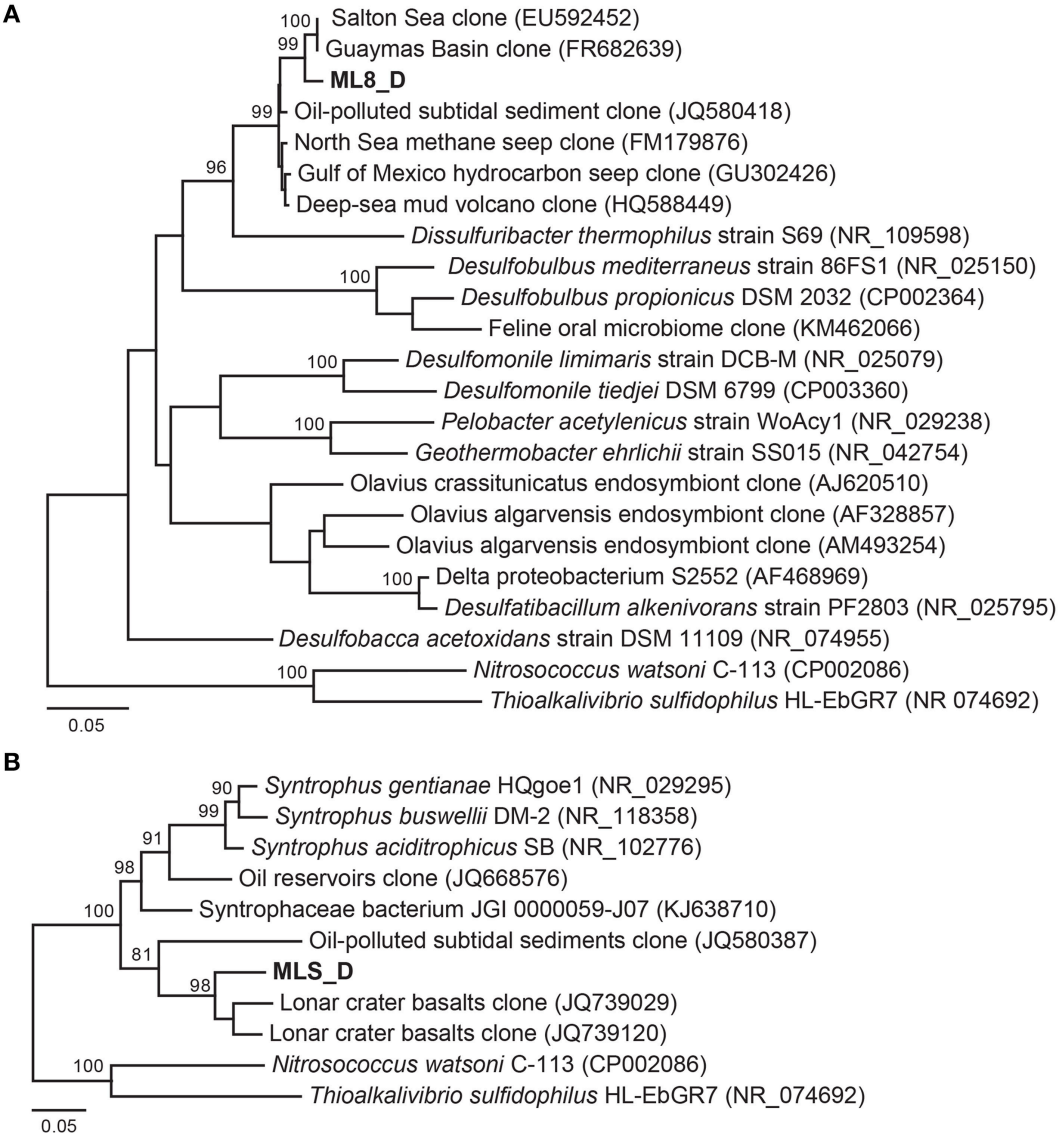
**Maximum likelihood based phylogenetic 16S rRNA gene tree of closely related Deltaproteobacteria and the Deltaproteobacteria genomic bins from the 8 m metagenome (A) and the sediment metagenome (B)**. Bootstrap support values based on 1000 bootstrap samplings >90 are noted.

Previous work has shown that a significant portion of the sulfide used by the dense layer of photoautotrophs in the ML chemocline at 7 m is generated by sulfate reduction within the chemocline (Overmann et al., 1991, 1996a). However, sulfide flux from below the chemocline has also been observed (Overmann et al., 1991) and sulfide concentrations increase with depth suggesting sulfate reducing bacteria are active below the chemocline (Northcote and Hall, 1983; Overmann et al., 1991). In addition, analysis of 16S rRNA sequences from ML are consistent with sulfate reduction in the hypolimnion and sediments (Klepac-Ceraj et al., 2012). Here, we reconstructed genomes of two Deltaproteobacteria species from the aphotic zone of ML—one from the 8-m sample and one from the sediments. According to phylogenetic analysis of 16S rRNA gene sequences and 18 concatenated ribosomal proteins in the bins (Figure 5A; Figure S3), the ML8_D population is closely related to uncultured deep-sea sediment clones and to *Dissulfuribacter thermophilus*, a hydrothermal vent chemolithoautotroph that disproportionates elemental sulfur to thiosulfate and sulfite (Figure 5A; Slobodkin et al., 2013). The ML8_D and *D. thermophilus* 16S rRNA sequences share 90% sequence identity. The closest relatives with fully sequenced genomes are *Desulfobulbus* spp. (Figure S3). The ML8_D genome is 98% complete and contains ~3.5 Mbp over 93 scaffolds with an average G+C content of 45.2%. The genome encodes a complete dissimilatory sulfate reduction pathway—a sulfate adenylyltransferase (ATP-sulfurylase, Sat), an APS reductase (AprAB), and the dissimilatory sulfite reductase complex (DsrAB, DsrC, and DsrMKJOP). In addition, the genome encodes all of the proteins necessary for dissimilatory nitrate reduction—nitrate reductase (Nar) and nitrite reductase (Nir) as well as genes encoding proteins for gluconeogenesis, ATP synthase, acetyl-CoA synthetase (the key enzyme for the utilization of acetate), some genes encoding homologs of TCA cycle proteins, and genes encoding flagella. The genome also encodes an acetyl-CoA synthase, a carbon monoxide dehydrogenase, a formate dehydrogenase and a Hox [NiFe]-hydrogenase. The presence of hydrogenase and formate dehydrogenase suggest that H_2_ and formate play important roles in the flow of electrons during sulfate reduction by the ML8_D population. However, the exact function of the NAD^+^-reducing hydrogenase (Hox) is not known—it has been suggested to play a role in removing excess electrons from fermentation (Horch et al., 2012). Some SRBs use CO dehydrogenase/acetyl-CoA synthase to fix carbon via the reductive acetyl-CoA pathway (Schauder et al., 1989); however, the ML8_D genome does not encode the full pathway. Although the genome is incomplete, the gene content for the ML8_D population suggests that these organisms are heterotrophic, motile, and capable of coupling anaerobic acetate oxidation to the reduction of sulfate.

Analysis of the Deltaproteobacteria bin from the sediments, MLS_D, indicates this population is related to clones from oil-impacted sediments and basalts and to previously characterized members of the genus *Syntrophus*, including the model organism *S. aciditrophicus* (Figure 5B; Figure S3). The MLS_D and *S. aciditrophicus* 16S rRNA sequences share 91% sequence identity. *S. aciditrophicus* degrades fatty acids and aromatic acids in syntrophic association with hydrogen/formate-using microorganisms (Jackson et al., 1999). MLS_D contained ~2.5 Mbp over 70 scaffolds with an average G+C content of 55.9%. The genome is 98% complete based on the presence of single copy marker genes (Table S2). The genome encodes the genetic machinery necessary for assimilatory sulfate reduction (the Cys system) as well as Nap (a periplasmic nitrate reductase), the genetic machinery necessary for assimilation of ammonia, and biosynthesis of flagella. The genes necessary for an F-type ATPase were also present as were the gluconeogenic and pentose phosphate pathway genes to synthesize hexose- and pentose-phosphates from acetyl-CoA. Genes encoding two of the subunits for the ATP-dependent, benzoyl-CoA reductase were detected as well as genes encoding an Rnf-type, ion-translocating, electron transport complex, and genes for transporters for uptake of branched-chain amino acids. Genes encoding multiple alcohol deyhrogenases were encoded in the MLS_D genome, including one that is closely related to *adh1* of *Desulfovibrio vulgaris* Hildenborough. In *D.vulgaris* Hildenborough, *adh1* is highly expressed in cells grown on lactate, pyruvate, formate, or hydrogen as electron donors for sulfate reduction (Haveman et al., 2003). Genes encoding proton-translocating, NADH:ubiquinone oxidoreductase (complex I) or succinate dehydrogenase (complex II) were not recovered. In addition, MLS_D genome did not contain the genes that would permit the use of external electron acceptors such as oxygen, nitrate, sulfate, iron, or organic molecules. While these genes are also lacking from the genome of the obligate syntroph *S. aciditrophicus*, the MLS_D genome is incomplete (~98% complete), and thus we cannot conclude that the MLS_D population in Mahoney Lake relies on hydrogen/formate-using microorganisms.

#### Firmicutes

**Figure 6:**
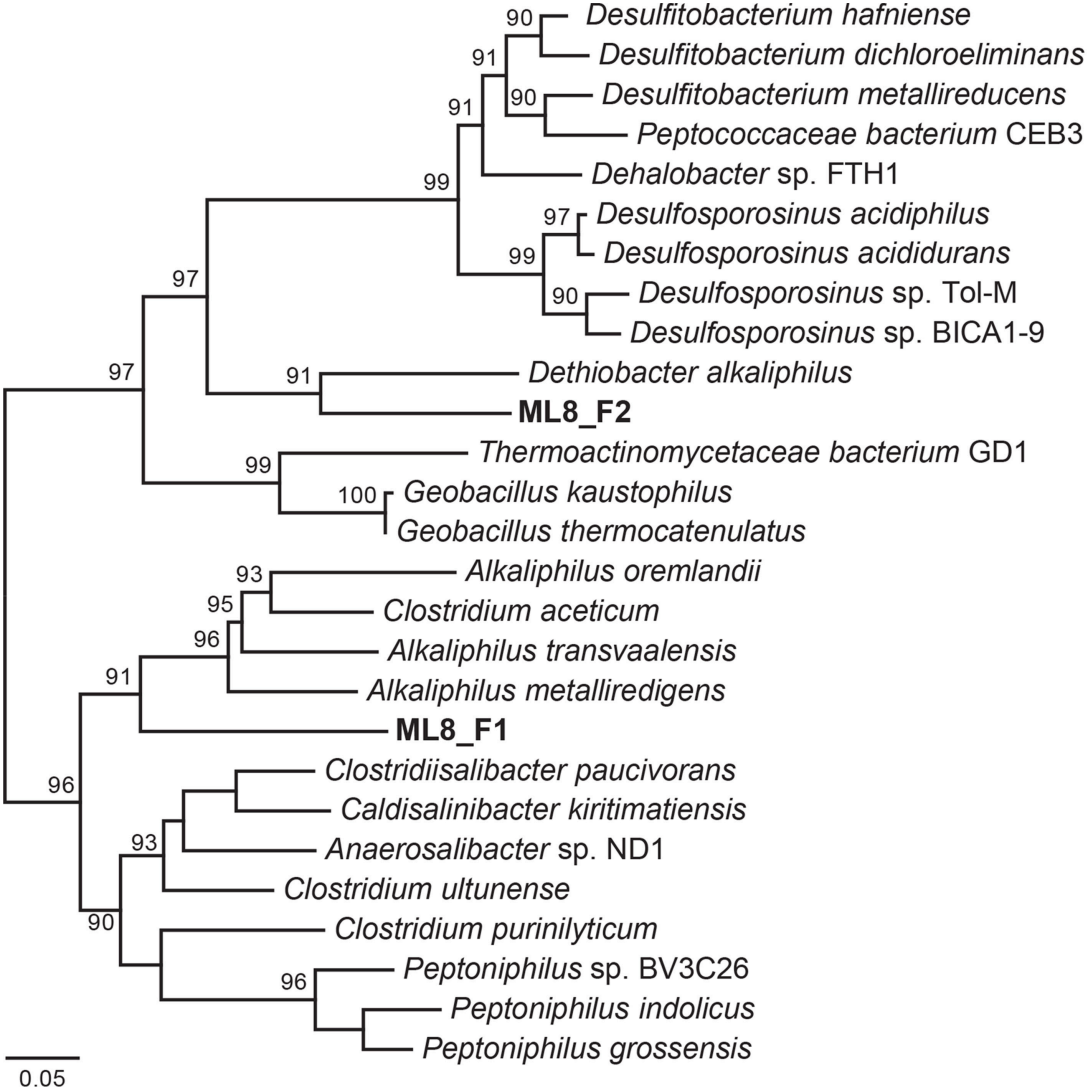
**Maximum likelihood phylogenetic tree of 18 concatenated single-copy ribosomal proteins (Table S3) the Clostridia species genomic bins**. Bootstrap support values based on 1000 bootstrap samplings >90 are noted.

We reconstructed two Firmicutes genomes from the 8-m sample. Based on phylogenetic analysis of 18 concatenated ribosomal proteins (Figure 6), the ML8_F1 population is most closely related to members of the *Alkaliphilus* genus, and ML8_F2 is most closely related to *D. alkaliphilus*. The ML8_F1 genome contained ~2.3 Mbp over 58 scaffolds with an average G+C content of 50.2% (Table 1). The ML8_F2 genome contained ~3.0 Mbp over 143 scaffolds with an average G+C content of 46.6%. Based on the presence of single copy phylogenetic marker genes (Table S2), the ML8_F1 genome is 98% complete while the ML8_F2 genome is 96% complete.

**Table 1:** **Statistics for the genome bins**.

	**8 m**			**Sediments**	
	**ML8_D**	**ML8_F1**	**ML8_F2**	**MLS_D**	**MLS_C**
Scaffolds	93	58	143	70	35
Longest scaffold (bp)	168,842	200,011	205,602	218,332	335,466
**GENERAL INFORMATION**					
Total bp	3,568,747	2,376,371	3,019,424	2,512,366	2,978,745
N50 (bp)	69,455	101,095	46,500	69,790	208,158
**CHARACTERISTICS**					
G + C	45.2	50.2	46.6	55.9	59.6
Protein coding genes	3112	3853	2272	2539	2726
Average coverage	27	23	12	21	31
% complete^a^	98	98	96	98	98

a *Based on the presence of single copy marker genes (Table S2)*.

The ML8_F1 genome is most closely related to *Alkaliphilus* spp.—including *A. metalliredigens, A. transvaalensis*, and *A. oremlandii. A. metalliredigens* was isolated from a leachate pond and is a metal-reducing alkaliphile (Ye et al., 2004). *A. oremlandii* can use thiosulfate as an electron acceptor with a variety of organic electron donors, including acetate, pyruvate, and formate (Fisher et al., 2008). The ML8_F2 genome is most closely related to *D. alkaliphilus*. In addition to sulfur disproportionation, *D. alkaliphilus* oxidizes H_2_ and is a facultative autotroph (Sorokin et al., 2008). The ML8_F1 genome encodes a Nuo-type NADH hydrogenase and an F-type ATPase. The ML8_F2 genome encodes components of a Nuo-type NADH dehydrogenase, a V-type ATPase, and an F-type ATPase. The ML8_F2 genome encodes a homolog of acetyl-CoA synthase acetyl-CoA synthase and a carbon monoxide dehydrogenase as well as all of the genes necessary to carry out this activity (Ragsdale and Pierce, 2008). The ML8_F1 genome encodes a carbon monoxide dehydrogenase but not the acetyl-CoA synthase. Both genomes encoded multiple [FeFe]-hydrogenases and one [NiFe]-hydrogenase—plus all the necessary hydrogenase maturation genes. Genes encoding enzymes necessary for assimilatory (Cys) and dissimilatory sulfate reduction (Dsr), as well as nitrogen fixation, denitrification, or nitrate reduction, were absent in both ML8_F genomes which are both >95% complete based on the presence of single copy marker genes. Genes encoding proteins involved in glycolysis and the tricarboxylic acid (TCA) cycle were present in both genomes, along with numerous transporters, particularly metal transporters. While further data are required to test our hypothesis that the ML8_F1 and F2 populations are acetogenic bacteria that use CO or CO_2_ plus H_2_ as their sole carbon and energy sources (Drake et al., 1994) our data do suggest a role for Firmicutes populations in hydrogen oxidation and acetate production in Mahoney Lake.

#### MLS_C genome

**Figure 7:**
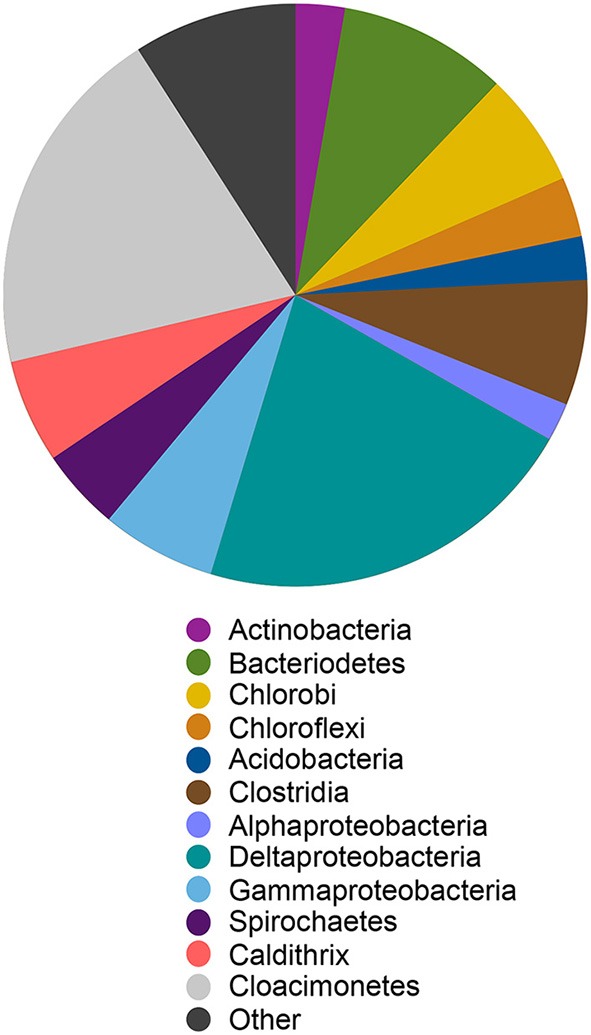
**Best BlastP hits for all predicted protein sequences in the Candidatus Aegiribacteria MLS_C genome**. Other indicates all phyla represented by < 5% of the total sequences in the genome. The relative abundance of taxonomic groups is summed at the phylum level except for Proteobacteria and Firmicutes which are summed at the class level and Caldithrix which are summed at the genus level.

We reconstructed a genome from the ML sediment metagenome that contains ~2.9 Mbp over 35 scaffolds with an average G+C content of 59.6%. The genome is nearly complete, encoding 98% of single copy phylogenetic marker genes (Table S2). The 16S rRNA gene sequence is most closely related to sequences recovered from Yuncheng Salt Lake of Shanxi Province, China which is rich in sulfate, chloride and magnesium; submarine methane seeps (Beal et al., 2009; Pachiadaki et al., 2010); and Zodletone Spring (anaerobic spring rich in methane, sulfide, and sulfur in Oklahoma, USA; Elshahed et al., 2007) as well as to the isolates *Caldithrix abyssi* and *Caldithrix paleocroryensis*, which are thermophilic, mixotrophic anaerobes from hydrothermal deep-sea environments (Miroshnichenko et al., 2003, 2010). The MLS_C 16S rRNA sequence shares only 83 and 84% sequence identity with the *C. abyssi* and *C. paleocroryensis* 16S rRNA sequences, respectively. In addition, the MLS_C 16S rRNA sequence shares 79% sequence identity with the 16S rRNA sequence from *Fibrobacter succinogenes* S85, a cellulolytic luminal mesophilic bacterium (Weimer, 1993). The predicted MLS_C proteins shared the highest percent sequence identity to proteins from many different phyla (Figure 7). The majority were affiliated with Deltaproteobacteria and *Candidatus* Cloacimonetes (WWE1 clade). Most *Caldithrix* sequences have been recovered from sulfide-rich marine niches, such as those found near hydrothermal vents, but they have also been recovered from mangrove soil, sulfidic caves, farm soil, methanogenic granular sludges, and in a benzoate-degrading consortium (Huang et al., 2010; Zhou et al., 2011; Alauzet and Jumas-Bilak, 2014). Sequences affiliated with *Candidatus* Cloacimonetes were first recovered from a wastewater treatment plant (Pelletier et al., 2008). Analysis of the single copy marker genes in these related isolates was similar—the highest percent sequence identity of individual proteins were from many different phyla, even though the majority of these sequences are on the same contig (Table S5).

**Figure 8:**
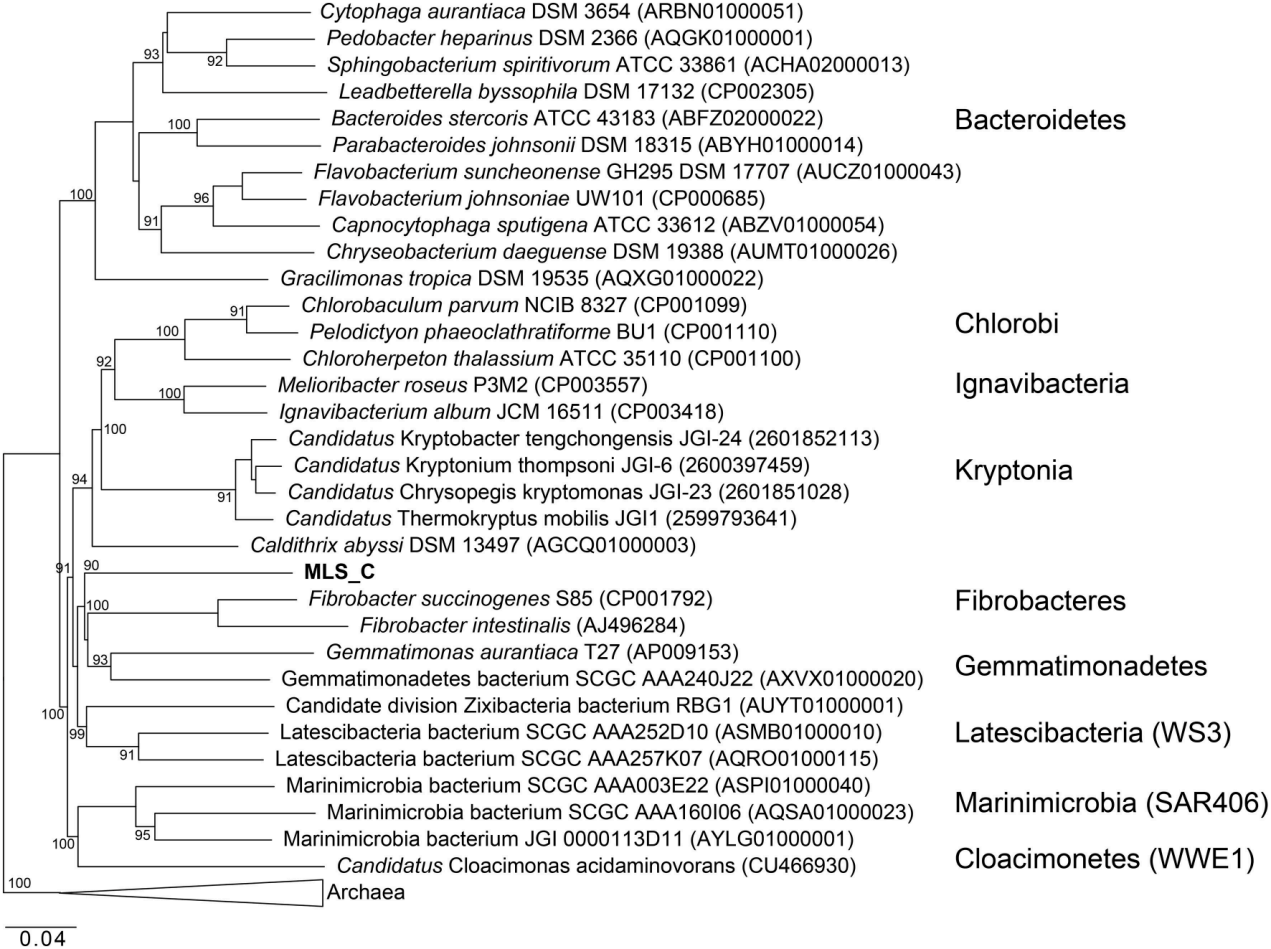
**Maximum likelihood based phylogenetic 16S rRNA gene tree of members of the FCB superphylum and MLS_C**. Bootstrap support values based on 1000 bootstrap samplings >90 are noted. NCBI accession numbers or IMG gene ids are shown in parentheses.

Phylogenetic analysis of the MLS_C 16S rRNA gene places the lineage into a monophyletic branch within the FCB superphylum MLS_C represents a deep-branching, monophyletic lineage (90% bootstrap support) with *F. succinogenes* S85 as the next relative (Figure 8). Phylogenetic analysis of the rpS3 sequences places the MLC_S lineage into a monophyletic branch (95% bootstrap support) with *Candidatus* Cloacimonetes acidaminovorans (Figure S4). The highest ANI observed in this limited data set was between the MLS_C genome and a Marinimicrobia bacterium genome (76.9%) whereas the average nucleotide identity between the MLS_C genome and the genomes of *F. succinogenes and Candidatus* Cloacimonetes acidaminovorans was 64.3 and 62.4%, respectively (Figure S4). Members of the clade Marinimicrobia (formally SAR406; Rinke et al., 2013) are common in marine environments (Yilmaz et al., 2015). Based on these data, the MLS_C population appears to be only distantly related to the *Caldithrix* genus, *Candidatus* Cloacimonetes, and *F. succinogenes* S85. Because the 16S rRNA gene from MLS_C shares low sequence identity (79–84%) to described bacterial phyla, and the sequence represents a deep-branching, monophyletic lineage, we propose that it represents a new genus within the FCB superphylum. In agreement with recently proposed guidelines for taxonomic classification of environmentally-derived genome sequences with currently accepted nomenclature standards (Hedlund et al., 2015), we propose the name *Candidatus* Aegiribacteria MLS_C after the Norse God of the Sea, Aegir, a renowned undersea brewer, reflecting its presumed fermentative metabolism. The FCB superphylum now includes the original phyla Fibrobacteres, Chlorobi, Bacteroidetes, and Ignavibacteriae as well as the candidate phyla Cloacimonetes (WWE1), Marinimicrobia (SAR406), Latescibacteria (WS3), Gemmatimonadetes, Hydrogenendentes (NKB19) (Rinke et al., 2013), the Zixibacteria RBG1 (Castelle et al., 2013), the recently discovered “Candidatus Kryptonia” (Eloe-Fadrosh et al., 2016), the *Caldithrix* genus and the new genus described here, *Candidatus* Aegiribacteria MLS_C.

The G+C content of the MLS_C genome is elevated (59.6%) compared to the *C. abyssi* genome (43.3%) and the *C. palaeochoryensis* genome (42.5%), as well as the reconstructed *Candidatus* Cloacamonas acidaminovorans genome (37.9%; Pelletier et al., 2008) and the *F. succinogenes* S85 genome (48%; Suen et al., 2011). There are only two characterized representatives of the *Caldithrix* genus, *C. abyssi*, and *C. palaeochoryensis*. *C. abyssi* oxidizes molecular hydrogen and acetate in the presence of nitrate, while *C. palaeochoryensis* is an obligately fermentative chemoorganotroph that can grow on di- and polysaccharides, as well as proteinaceous substrates (Alauzet and Jumas-Bilak, 2014). *Ca*. Cloacimonetes sequences have been recovered from anaerobic digesters (Pelletier et al., 2008; Lykidis et al., 2011; Wu et al., 2013; Limam et al., 2014; Nobu et al., 2015b). *Ca*. Cloacimonetes have been inferred to perform syntrophic propionate degradation (Nobu et al., 2015b) and the anaerobic digestion of cellulose (Limam et al., 2014), but no isolates have been fully characterized. Selective enrichment and a reconstructed genome sequence of *Ca.* Cloacamonas acidaminovorans suggest the organism is syntrophic (Pelletier et al., 2008). *F. succinogenes* S85 produces succinate, acetate, and formate as major fermentative end products and is specialized to derive energy from cellulose (Weimer, 1993; Suen et al., 2011).

Genes encoding pili and flagella were present in the ML_C genome suggesting that MLS_C is motile. Genes encoding a periplasmic [FeFe]-hydrogenase and a [NiFe]-hydrogenase and all necessary hydrogenase maturation genes were present, as well as a heterodisulfide reductase that is widespread in anaerobic bacteria. The genome encodes homologs of D-lactate dehydrogenase (gene *ldhA*) and pyruvate dehydrogenase (gene *pdh*) for conversion of lactate to acetyl-CoA, as well as carbon monoxide dehydrogenase/acetyl-CoA synthetase (CODH/ACS). The MLS_C genomes is nearly complete (~98%) and similar to the *Ca.* Cloacamonas acidaminovorans genome, the genome does not contain genes encoding the enzymes necessary to synthesize certain amino acids. The genome encodes all of the machinery necessary for degradation of organic matter for acquisition of carbon and nitrogen including multiple proteases and peptidases. The genome also encodes the proteins necessary for the oxidation of propionate into acetate and carbon dioxide via methylmalonyl-CoA, succinate, fumarate, malate, oxaloacetate, pyruvate, and acetyl-CoA as intermediates. This pathway is found in obligate syntrophic bacteria (Schink, 1997) and is also encoded in the *Ca.* Cloacamonas acidaminovorans genome (Pelletier et al., 2008). This pathway is only thermodynamically favorable under low H_2_ conditions, such as in syntrophic consortia with H_2_-scavenging bacteria (Megonigal et al., 2004; Ariesyady et al., 2007). Populations capable of scavenging H_2_, such as sulfate reducing bacteria and acetogenic bacteria, are present in the sediment metagenome and could fulfill this partnership.

### Carbon and sulfur cycling in Mahoney Lake

**Figure 9:**
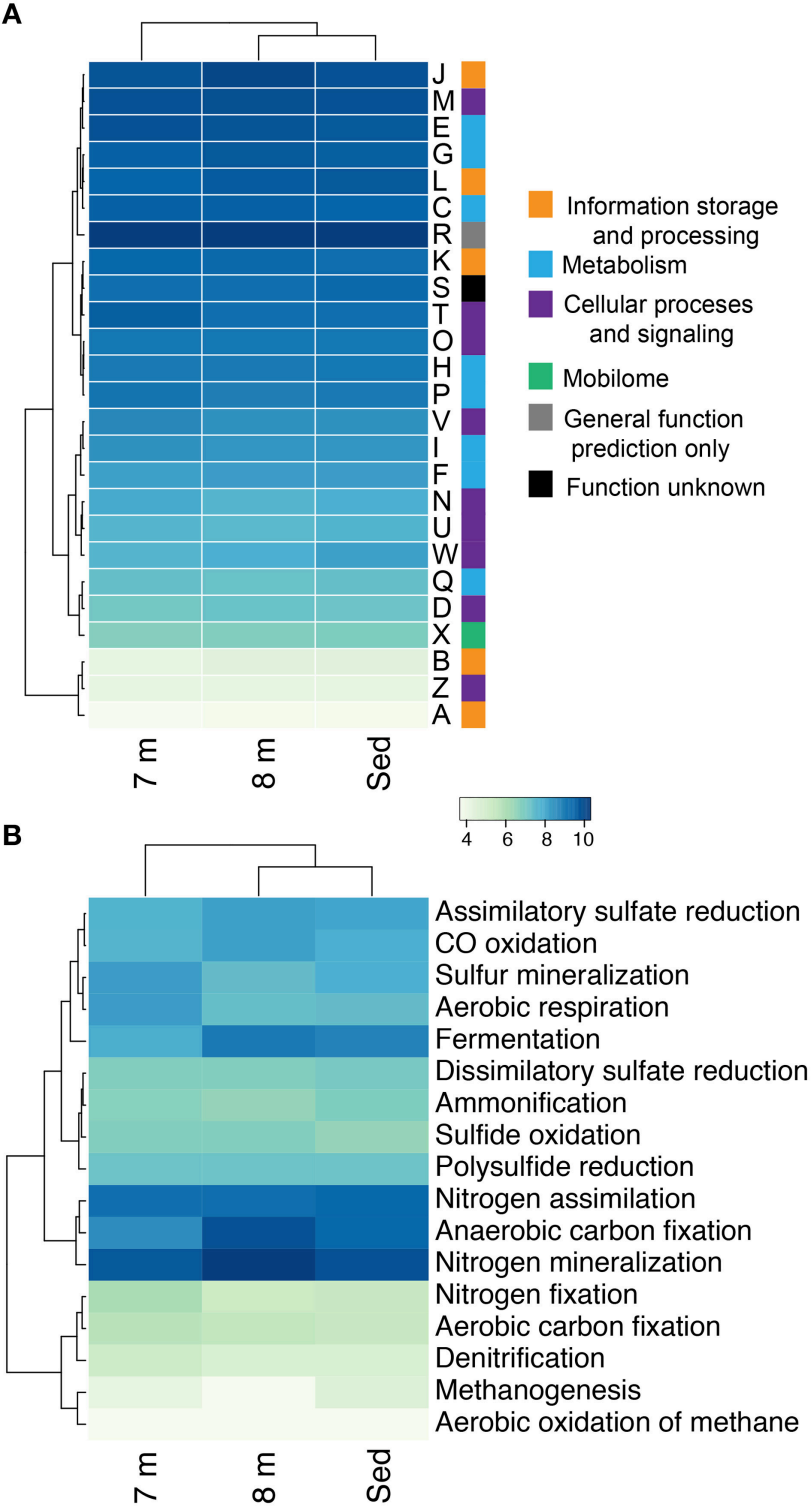
**Hierarchical clustering of the relative abundance of COG categories (A) and the genetic potential for carbon, nitrogen, and sulfur cycling in each assemblage at 7, 8 m, and in the sediments of Mahoney Lake (B)**. Values within each category are normalized across samples (see Section Materials and Methods). Clustering analyses is based on the normalized abundance profiles of COGs. The genetic potential for each step in **(B)** was estimated using a combination of normalized marker gene ratios as previously described (Lauro et al., 2011). Marker genes are provided in Table S4. The 7 m data set is from Hamilton et al. (2014). Sed, Sediment; A, RNA processing and modification; B, chromatin structure and dynamics; C, energy production and conversion; D, cell cycle control, cell division, chromosome partitioning; E, amino-acid transport and metabolism; F, nucleotide transport and metabolism; G, carbohydrate transport and metabolism; H, coenzyme transport and metabolism; I, lipid transport and metabolism; J, translation, ribosomal structure, and biogenesis; K, transcription; L, replication, recombination, and repair; M, cell wall/membrane/envelope biogenesis; N, cell motility; O, post-translational modification, protein turnover, chaperones; P, inorganic ion transport and metabolism; and Q, secondary metabolites biosynthesis, transport, and catabolism; R, general function prediction only; S, function unknown; T, signal transduction mechanisms; U, intracellular trafficking, secretion, and vesicular transport; W, extracellular structures; X, nuclear structure; V, defense mechanisms; Z, cytoskeleton.

16S rRNA sequencing indicated that different layers of ML are host to distinct microbial assemblages (Klepac-Ceraj et al., 2012). Our metagenomic sequencing suggests these microbial assemblages are similar phylogenetically at the phylum or class level (Figure 1) despite marked differences in geochemistry throughout the stratified water column—the epilimnion is oxic and oligotrophic whereas the hypolimnion is anoxic, more saline, eutrophic and rich in sulfate, sulfide, and polysulfide (Hall and Northcote, 1986). To assess potential differences in functional diversity at the chemocline and below, we compared COG functional gene assignments between the 7-m (chemocline), 8-m, and sediment communities. These data indicate similar COG distributions in assemblages from the three horizons (Figure 9A; Figure S5A). COGs represented at high abundance included common functions such as translation, ribosomal structure, and biogenesis; energy production and conversion; and replication, recombination, and repair. The sediment and 8-m communities contain a more similar distribution of COG categories compared to the 7-m community. Because this analysis is based on COG categories, we examined the 50 most abundant COGs to determine what functional differences might be detectable between the horizons. In this analysis, the 7- and 8-m communities were more similar than the sediment community (Figure S5B). Putative proteins with lower relative abundance in the 7- and 8-m assemblages compared to the sediments included COG0840 and COG1372. COG0840 is involved in signal transduction and cell motility, while COG1372 is an endonuclease with a role in DNA replication, recombination, and repair. Sequences with lower relative abundance in the sediments included proteins involved in signal transduction (COG0745) and DNA replication, recombination, and repair (COG4974). COG1595, a DNA-directed RNA polymerase, had a higher relative abundance at 7 and 8 m compared to the sediments. A putative NAD(P)-dependent dehydrogenase (COG1028) was also more abundant in and near the chemocline.

Our recent analysis of the Mahoney Lake chemocline indicated functional redundancy in both the oxidative and reductive arms of the sulfur cycles within the narrow phototrophic plate at 7 m (Hamilton et al., 2014). Comparison of COGs from 7, 8 m, and the sediments reveals no difference in the abundance of assimilatory or dissimilatory sulfate reduction, sulfur mineralization, or polysulfide reduction. These data suggest that similar processes are also active below the chemocline or that our data include genetic material that settled from the chemocline (Figure 9B). In the COG analysis, the marker genes for dissimilatory sulfate reduction and sulfide oxidation (K00394, K00395, K00396) are equivalent and can operate in either process. In the 8-m and sediment metagenomes, the majority of sequences cluster with those involved in dissimilatory sulfate reduction, consistent with recovery of multiple populations capable of reducing oxidized sulfur below the chemocline (data not shown). In contrast, the majority of the same COGs from the 7-m metagenome clustered with sequences involved in sulfur oxidation (Hamilton et al., 2014). The hypolimnion (8 m) and sediments are also rich in polysulfides, elemental sulfur, and other sulfur intermediates that can be disproportionated (Bak and Cypionka, 1987; Finster, 2008; Poser et al., 2013). We observed multiple populations capable of reducing oxidized sulfur compounds in the highly sulfidic (35 mM) hypolimnion but very little evidence for sulfur oxidation. Autotrophic growth by sulfite/thiosulfate disproportionation appears to be a common trait among haloalkaliphilic SRB (Sorokin et al., 2011). Based on the recovery of taxonomic markers genes (16S rRNA and rpS3 sequences) related to Deltaproteobacteria that disproportionate sulfur compounds and the abundance of sulfur cycle intermediates below the chemoline in Mahoney Lake, it is plausible that disproportionation is an important process in ML.

The redox stability of ML can be attributed to its geochemistry—that is, high salinity and high sulfur concentrations—and a physiographic position that results in minimal wind mixing and the delivery of allochthonous carbon required to supply the extra flux of electrons needed to sustain sulfate reduction (Overmann, 1997). Oxygenic primary production in the epilimnion is not sufficient to supply electrons for sulfate reduction in the form of phytoplanktonic carbon fixed *in situ*—instead, organic matter is supplied from the surrounding landscape (Overmann et al., 1996a,b; Overmann, 1997). The strong density stratification and the related redox profile help retain organic matter in neutrally buoyant layers, supporting remineralization of organic matter in the water column (Overmann, 1997)—a feature that is also common in some marine systems (MacIntyre et al., 1995; Sorokin, 2002). ML hosts one of the densest chemocline phototroph communities observed to date, yet direct export of this planktonic organic matter from the chemocline to the sediments is low (Overmann, 1997). Based on fatty acid profiles and bulk organic carbon isotopes, organic matter from the water column is distinguishable from sediment organic matter (Bovee and Pearson, 2014). The latter has characteristics dominantly of allochthonous, higher-plant sources and is most likely sourced from the margins of the lake basin. In contrast, most of the planktonic organic matter is lost from the shallow water column through respiration. We found abundant evidence for anaerobic respiration below the chemocline, while proteins necessary for aerobic respiration were elevated at 7 m compared to 8 m and the sediments (Figure 9B).

In this study we recovered abundant evidence for heterotrophic—and especially fermentative—organisms at 8 m and in the sediments. The relative abundance of sequences for proteins putatively involved in anaerobic carbon fixation and fermentation were elevated relative to the chemocline (Figure 9B). Our data also indicate the presence of syntrophic populations and heterotrophs capable of degrading cellulose in the sediments. These observations are consistent with intense organic matter remineralization in the water column and delivery of more complex carbon substrates to the sediments.

## Conclusion

Mahoney Lake is an extreme meromictic system with unusually high levels of sulfate and sulfide. Here, we observed diverse microbial assemblages that are taxonomically similar at the phylum level yet functionally distinct in different layers of the lake. Metagenomic sequencing recovered abundant anaerobic bacteria from below the chemocline that are putatively involved in remineralizing organic matter and genes encoding for hydrogenases presumably due to organic matter loading and the need to dispose of excess electrons. Although the precise biochemical pathway of sulfur disproportionation remains uncharacterized, we recovered sequences most closely related to Deltaproteobacteria spp. known to perform this reaction in pure cultures. These data, along with the abundance of zero valent sulfur below the chemocline (Overmann et al., 1996a), suggest a role for sulfur disproportionation in highly euxinic systems despite the observation that elemental sulfur disproportionation becomes thermodynamically unfavorable under high sulfide concentrations (Canfield and Thamdrup, 1994; Rabus et al., 2000). We recovered a breadth of taxonomic and functional diversity from below the chemocline in an extreme euxinic environment including a nearly complete genome of *Candidatus* Aegiribacteria MLS_C, a new genus within the FCB superphylum. Our data highlight the role of these diverse assemblages in maintaining euxinia and suggest undiscovered taxa and diversity exist in other dark, sulfur-rich environments.

## Author contributions

TH, WG, TL, AP, and JM designed the study. AP, WG, and TL collected the samples. TH, RB, SS, WM, and AP performed the analyses. TH, RB, SS, WM, WG, TL, AP, and JM contributed to writing, editing and finalizing the manuscript.

### Conflict of interest statement

The authors declare that the research was conducted in the absence of any commercial or financial relationships that could be construed as a potential conflict of interest.

## References

[B1] Alauzet C. Jumas-Bilak E. (2014). The phylum Deferribacteres and the genus Caldithrix, in The Prokaryotes – Other Major Lineages of Bacteria and the Archaea, eds Rosenbrug E. DeLong E. F. Lory S. Stackebrandt E. Thompson F. (Berlin; Heidelberg: Springer-Verlag), 595–611.

[B2] Amend J. P. Shock E. S. (2001). Energetics of overall metabolic reactions of thermophilic and hyperthermophilic Archaea and Bacteria. FEMS Microbiol. Rev. 25, 175–243. 10.1111/j.1574-6976.2001.tb00576.x 11250035

[B3] Ariesyady H. D. Ito T. Yoshiguchi K. Okabe S. (2007). Phylogenetic and functional diversity of propionate-oxidizing bacteria in an anaerobic digester sludge. Appl. Microbiol. Biotechnol. 75, 673–683. 10.1007/s00253-007-0842-y 17262205

[B4] Bak F. Cypionka H. (1987). A novel type of energy-metabolism involving fermentation of inorganic sulfur-compounds. Nature 326, 891–892. 10.1038/326891a0 22468292

[B5] Bak F. Pfennig N. (1987). Chemolithotrophic growth of *Desulfovibrio sulfodismutans* sp. nov. by disproportionation of inorganic sulfur compounds. Arch. Microbiol. 147, 184–189. 10.1007/BF00415282

[B6] Beal E. J. House C. H. Orphan V. J. (2009). Manganese- and iron-dependent marine methane oxidation. Science 325, 184–187. 10.1126/science.1169984 19589998

[B7] Bosshard P. P. Santini Y. Grüter D. Stettler R. Bachofen R. (2000). Bacterial diversity and community composition in the chemocline of the meromictic alpine Lake Cadagno as revealed by 16S rDNA analysis. FEMS Microbiol. Ecol. 31, 173–182. 10.1111/j.1574-6941.2000.tb00682.x 10640670

[B8] Bovee R. Pearson A. (2014). Strong influence of the littoral zone on sedimentary lipid biomarkers in a meromictic lake. Geobiology 12, 529–541. 10.1111/gbi.12099 25201322

[B9] Briée C. Moreira D. López-García P. (2007). Archaeal and bacterial community composition of sediment and plankton from a suboxic freshwater pond. Res. Microbiol. 158, 213–227. 10.1016/j.resmic.2006.12.012 17346937

[B10] Brocks J. J. Love G. D. Summons R. E. Knoll A. H. Logan G. A. Bowden S. A. (2005). Biomarker evidence for green and purple sulphur bacteria in a stratified Palaeoproterozoic sea. Nature 437, 866–870. 10.1038/nature04068 16208367

[B11] Brown C. T. Hug L. A. Thomas B. C. Sharon I. Castelle C. J. Singh A.. (2015). Unusual biology across a group comprising more than 15% of domain Bacteria. Nature 523, 208–2011. 10.1038/nature14486 26083755

[B12] Bryant M. P. Campbell L. L. Reddy C. A. Crabill M. R. (1977). Growth of *Desulfovibrio* in lactate or ethanol media low in sulfate in association with H_2_-utilizing methanogenic bacteria. Appl. Environ. Microbiol. 33, 1162–1169. 879775 10.1128/aem.33.5.1162-1169.1977 PMC170843

[B13] Canfield D. E. (1998). A new model for Proterozoic ocean chemistry. Nature 396, 450–453. 10.1038/24839

[B14] Canfield D. E. (2013). Sulfur isotopes in coal constrain the evolution of the Phanerozoic sulfur cycle. Proc. Natl. Acad. Sci. U.S.A. 110, 8443–8446. 10.1073/pnas.1306450110 23650346 PMC3666666

[B15] Canfield D. E. Thamdrup B. (1994). The production of 34S-depleted sulfide during bacterial disproportionation of elemental sulfur. Science 266, 1973–1975. 10.1126/science.11540246 11540246

[B16] Carr S. A. Orcutt B. N. Mandernack K. W. Spear J. R. (2015). Abundant atribacteria in deep marine sediment from the Adélie Basin, Antarctica. Front. Microbiol. 6:872. 10.3389/fmicb.2015.00872 26379647 PMC4549626

[B17] Castelle C. J. Hug L. A. Wrighton K. C. Thomas B. C. Williams K. H. Wu D.. (2013). Extraordinary phylogenetic diversity and metabolic versatility in aquifer sediment. Nat. Commun. 4, 2120. 10.1038/ncomms3120 23979677 PMC3903129

[B18] Castelle C. J. Wrighton K. C. Thomas B. C. Hug L. A. Brown C. T. Wilkins M. J.. (2015). Genomic expansion of domain Archaea highlights roles for organisms from new Phyla in anaerobic carbon cycling. Curr. Biol. 25, 690–701. 10.1016/j.cub.2015.01.014 25702576

[B19] Coolen M. J. L. Overmann J. (1998). Analysis of subfossil molecular remains of purple sulfur bacteria in a Lake Sediment. Appl. Environ. Microbiol. 64, 4513–4521. 9797316 10.1128/aem.64.11.4513-4521.1998 PMC106678

[B20] Darriba D. Taboada G. Doallo R. Posada D. (2012). jModelTest 2: more models, new heuristics and parallel computing. Nat. Methods 9, 772. 10.1038/nmeth.2109 22847109 PMC4594756

[B21] Decristophiris P. M. A. Peduzzi S. Ruggeri-Bernardi N. Hahn D. Tonolla M. (2009). Fine scale analysis of shifts in bacterial community structure in the chemocline of meromictic Lake Cadagno, Switzerland. J. Limnol. 68, 16–24. 10.4081/jlimnol.2009.16

[B22] Del Don C. Hanselmann K. W. Peduzzi R. Bachofen R. (2001). The meromictic alpine Lake Cadagno: orographical and biogeochemical description. Aquat. Sci. 63, 70–90. 10.1007/PL00001345

[B23] Delmont T. O. Malandain C. Prestat E. Larose C. Monier J. M. Simonet P.. (2011). Metagenomic mining for microbiologists. ISME J. 5, 1837–1843. 10.1038/ismej.2011.61 21593798 PMC3223302

[B24] Dick G. J. Andersson A. F. Baker B. J. Simmons S. L. Thomas B. C. Yelton A. P.. (2009). Community-wide analysis of microbial genome sequence signatures. Genome Biol. 10:R85. 10.1186/gb-2009-10-8-r85 19698104 PMC2745766

[B25] Di Rienzi S. C. Sharon I. Wrighton K. C. Koren O. Hug L. A. Thomas B. C.. (2013). The human gut and groundwater harbor non-photosynthetic bacteria belonging to a new candidate phylum sibling to Cyanobacteria. Elife. 2:e01102. 10.7554/eLife.01102 24137540 PMC3787301

[B26] Dodsworth J. A. Blainey P. C. Murugapiran S. K. Swingley W. D. Ross C. A. Tringe S. G.. (2013). Single-cell and metagenomic analyses indicate a fermentative and saccharolytic lifestyle for members of the OP9 lineage. Nat. Commun. 4, 1854. 10.1038/ncomms2884 23673639 PMC3878185

[B27] Drake H. L. Daniel S. L. Matthies C. Küsel K. (1994). Acetogenesis, acetogenic bacteria, and the acetyl-CoA pathway: past and current perspectives, in Acetogenesis, ed Drake H. L. (New York, NY: Chapman & Hall), 3–60.

[B28] Durbin R. Eddy S. Krogh A. Mitchison G. (1998). Biological Sequence Analysis: Probabilistic Models of Proteins and Nucleic Acids. Cambridge, UK: Cambridge University Press.

[B29] Eady R. R. (1996). Structure-function relationships of alternative nitrogenase. Chem. Rev. 96, 3013–3030. 10.1021/cr950057h 11848850

[B30] Edgar R. C. Haas B. J. Clemente J. C. Quince C. Knight R. (2011). UCHIME improves sensitivity and speed of chimera detection. Bioinformatics. 27, 2194–2200. 10.1093/bioinformatics/btr381 21700674 PMC3150044

[B31] Eloe-Fadrosh E. A. Paez-Espino D. Jarett J. Dunfield P. F. Hedlund B. P. Dekas A. E.. (2016). Global metagenomic survey reveals a new bacterial candidate phylum in geothermal springs. Nat. Commun. 7:10476. 10.1038/ncomms10476 26814032 PMC4737851

[B32] Elshahed M. S. Youssef N. H. Luo Q. Najar F. Z. Roe B. A. Sisk T. M.. (2007). Phylogenetic and metabolic diversity of Planctomycetes from anaerobic, sulfide- and sulfur-rich Zodletone Spring, Oklahoma. Appl. Environ. Microbiol. 73, 4707–4716. 10.1128/AEM.00591-07 17545322 PMC1951033

[B33] Ferreira A. J. S. Siam R. Setubal J. C. Moustafa A. Sayed A. Chambergo F. S.. (2014). Core microbial functional activities in ocean environments revealed by global metagenomic profiling analyses. PLoS ONE 9:e97338. 10.1371/journal.pone.0097338 24921648 PMC4055538

[B34] Finster K. (2008). Microbial disproportionation of inorganic sulfur compounds. J. Sulfur Chem. 29, 281–292. 10.1080/17415990802105770

[B35] Fisher E. Dawson A. M. Polshyna G. Lisak J. Crable B. Perera E.. (2008). Transformation of inorganic and organic arsenic by *Alkaliphilus oremlandii* sp. nov. Strain OhILAs. Ann. N.Y. Acad. Sci. 1125, 230–241. 10.1196/annals.1419.006 18378595

[B36] Frey M. (2002). Hydrogenases: hydrogen-activating enzymes. ChemBioChem 3, 153–160. 10.1002/1439-7633(20020301)3:2/3&lt;153::AID-CBIC153&gt;3.0.CO;2-B 11921392

[B37] Gies E. A. Konwar K. M. Beatty J. T. Hallam S. J. (2014). Illuminating microbial dark matter in meromictic sakinaw lake. Appl. Environ. Microbiol. 80, 6807–6818. 10.1128/AEM.01774-14 25172853 PMC4249029

[B38] Gregersen L. H. Habicht K. S. Peduzzi S. Tonolla M. Canfield D. E. Miller M.. (2009). Dominance of a clonal green sulfur bacterial population in a stratified lake. FEMS Microbiol. Ecol. 70, 30–41. 10.1111/j.1574-6941.2009.00737.x 19656193

[B39] Grice K. Cao C. Q. Love G. D. Bottcher M. E. Twitchett R. J. Grosjean E.. (2005). Photic zone euxinia during the Permian-Triassic superanoxic event. Science 307, 706–709. 10.1126/science.1104323 15661975

[B40] Guindon S. Gascuel O. (2003). A simple, fast, and accurate algorithm to estimate large phylogenies by maximum likelihood. Syst. Biol. 52, 696–704. 10.1080/10635150390235520 14530136

[B41] Hall K. J. Northcote T. G. (1986). Conductivity-temperature standardization and dissolved solids estimation in a meromictic saline lake. Can. J. Fish. Aquat. Sci. 43, 2450–2454. 10.1139/f86-304

[B42] Hamilton T. L. Bovee R. J. Thiel V. Sattin S. R. Mohr W. Schaperdoth I.. (2014). Coupled reductive and oxidative sulfur cycling in the phototrophic plate of a meromictic lake. Geobiology 12, 451–468. 10.1111/gbi.12092 24976102

[B43] Harris J. K. Kelley S. T. Pace N. R. (2004). New perspectives on uncultured bacterial phylogenetic division OP11. Appl. Environ. Microbiol. 70, 845–849. 10.1128/AEM.70.2.845-849.2004 14766563 PMC348892

[B44] Haveman S. A. Brunelle V. Voordouw J. K. Voordouw G. Heidelberg J. F. Rabus R. (2003). Gene expression analysis of energy metabolism mutants of Desulfovibrio vulgaris Hildenborough indicates an important role for alcohol dehydrogenase. J. Bacteriol. 185, 4345–4353. 10.1128/JB.185.15.4345-4353.2003 12867442 PMC165767

[B45] Hedlund B. P. Dodsworth J. A. Staley J. T. (2015). The changing landscape of microbial biodiversity exploration and its implications for systematics. Syst. Appl. Microbiol. 38, 231–236. 10.1016/j.syapm.2015.03.003 25921438

[B46] Horch M. Lauterbach L. Lenz O. Hildebrandt P. Zebger I. (2012). NAD(H)-coupled hydrogen cycling – structure–function relationships of bidirectional [NiFe] hydrogenases. FEBS Lett. 586, 545–556. 10.1016/j.febslet.2011.10.010 22056977

[B47] Huang Y. Gilna P. Li W. (2009). Identification of ribosomal RNA genes in metagenomic fragments. Bioinformatics 25, 1338–1340. 10.1093/bioinformatics/btp161 19346323 PMC2677747

[B48] Huang Y. J. Kim E. Cox M. J. Brodie E. L. Brown R. Wiener-Kronish J. P.. (2010). A persistent and diverse airway microbiota present during chronic obstructive pulmonary disease exacerbations. OMICS 14, 9–59. 10.1089/omi.2009.0100 20141328 PMC3116451

[B49] Hug L. A. Castelle C. J. Wrighton K. C. Thomas B. C. Sharon I. Frischkorn K. R.. (2013). Community genomic analyses constrain the distribution of metabolic traits across the Chloroflexi phylum and indicate roles in sediment carbon cycling. Microbiome 1:22. 10.1186/2049-2618-1-22 24450983 PMC3971608

[B50] Huson D. H. Mitra S. Weber N. Ruscheweyh H. Schuster S. C. (2011). Integrative analysis of environmental sequences using MEGAN4. Genome Res. 21, 1552–1560. 10.1101/gr.120618.111 21690186 PMC3166839

[B51] Hyatt D. Chen G. L. Locascio P. F. Land M. L. Larimer F. W. Hauser L. J. (2010). Prodigal: prokaryotic gene recognition and translation initiation site identification. BMC Bioinformatics 11:119. 10.1186/1471-2105-11-119 20211023 PMC2848648

[B52] Imachi H. Sekiguchi Y. Kamagata Y. Loy A. Qiu Y.-L. Hugenholtz P.. (2006). Non-sulfate-reducing, syntrophic bacteria affiliated with *Desulfotomaculum* Cluster I are widely distributed in methanogenic environments. Appl. Environ. Microbiol. 72, 2080–2091. 10.1128/AEM.72.3.2080-2091.2006 16517657 PMC1393244

[B53] Inagaki F. Nunoura T. Nakagawa S. Teske A. Lever M. Lauer A.. (2006). Biogeographical distribution and diversity of microbes in methane hydrate-bearing deep marine sediments on the Pacific Ocean Margin. Proc. Natl. Acad. Sci. U.S.A. 103, 2815–2820. 10.1073/pnas.0511033103 16477011 PMC1413818

[B54] Jackson B. E. Bhupathiraju V. K. Tanner R. S. Woese C. R. McInerney M. J. (1999). *Syntrophus aciditrophicus* sp. nov., a new anaerobic bacterium that degrades fatty acids and benzoate in syntrophic association with hydrogen-using microorganisms. Arch. Microbiol. 171, 107–114. 10.1007/s002030050685 9914307

[B55] Johnston D. T. Wolfe-Simon F. Pearson A. Knoll A. H. (2009). Anoxygenic photosynthesis modulated Proterozoic oxygen and sustained Earth's middle age. Proc. Natl. Acad. Sci. U.S.A. 106, 16925–16929. 10.1073/pnas.0909248106 19805080 PMC2753640

[B56] Jones D. S. Albrecht H. L. Dawson K. S. Schaperdoth I. Freeman K. H. Pi Y.. (2012). Community genomic analysis of an extremely acidophilic sulfur-oxidizing biofilm. ISME J. 6, 158–170. 10.1038/ismej.2011.75 21716305 PMC3246232

[B57] Klepac-Ceraj V. Hayes C. A. Gilhooly W. P. Lyons T. W. Kolter R. Pearson A. (2012). Microbial diversity under extreme euxinia: Mahoney Lake, Canada. Geobiology 10, 223–235. 10.1111/j.1472-4669.2012.00317.x 22329601

[B58] Konstantinidis K. T. Braff J. Karl D. M. DeLong E. F. (2009). Comparative metagenomic analysis of a microbial community residing at a depth of 4,000 meters at station ALOHA in the North Pacific subtropical gyre. Appl. Environ. Microbiol. 75, 5345–5355. 10.1128/AEM.00473-09 19542347 PMC2725473

[B59] Lauro F. M. DeMaere M. Z. Yau S. Brown M. V. Ng C. Wilkins D.. (2011). An integrative study of a meromictic lake ecosystem in Antarctica. ISME J. 5, 879–895. 10.1038/ismej.2010.185 21124488 PMC3105772

[B60] Leavitt W. D. Halevy I. Bradley A. S. Johnston D. T. (2013). Influence of sulfate reduction rates on the Phanerozoic sulfur isotope record. Proc. Natl. Acad. Sci. U.S.A. 110, 11244–11249. 10.1073/pnas.1218874110 23733944 PMC3710818

[B61] Li H. Durbin R. (2009). Fast and accurate short read alignment with burrows-wheeler transform. Bioinformatics 25, 1754–1760. 10.1093/bioinformatics/btp324 19451168 PMC2705234

[B62] Limam R. D. Chouari R. Mazéas L. Wu T.-D. Li T. Grossin-Debattista J.. (2014). Members of the uncultured bacterial candidate division WWE1 are implicated in anaerobic digestion of cellulose. Microbiologyopen 3, 157–167. 10.1002/mbo3.144 24497501 PMC3996565

[B63] Llorens-Marès T. Yooseph S. Goll J. Hoffman J. Vila-Costa M. Borrego C. M.. (2015). Connecting biodiversity and potential functional role in modern euxinic environments by microbial metagenomics. ISME J. 9, 1648–1661. 10.1038/ismej.2014.254 25575307 PMC4478705

[B64] Lloyd K. G. Schreiber L. Peterson D. G. Kjeldson K. U. Lever M. A. Steen A. D.. (2013). Predominant Archaea in marine sediments degrade detrital proteins. Nature 496, 215–218. 10.1038/nature12033 23535597

[B65] Lohse M. Bolger A. M. Nagel A. Fernie A. R. Lunn J. E. Stitt M.. (2012). RobiNA: a user-friendly, integrated software solution for RNA-Seq-based transcriptomics. Nucleic Acids Res. 40, W622–W627. 10.1093/nar/gks540 22684630 PMC3394330

[B66] Lowe T. M. Eddy S. R. (1997). tRNAscan-SE: a program for improved detection of transfer RNA genes in genomic sequence. Nucleic Acids Res. 25, 955–964. 10.1093/nar/25.5.0955 9023104 PMC146525

[B67] Ludwig W. Strunk O. Westram R. Richter L. Meier H. Buchner A.. (2004). ARB: a software environment for sequence data. Nucleic Acids Res. 32, 1363–1371. 10.1093/nar/gkh293 14985472 PMC390282

[B68] Lukashin A. Borodovsky M. (1998). GeneMark.hmm: new solutions for gene finding. Nucleic Acids Res. 26, 1107–1115. 10.1093/nar/26.4.1107 9461475 PMC147337

[B69] Lykidis A. Chen C. L. Tringe S. G. McHardy A. C. Copeland A. Kyrpides N. C.. (2011). Multiple syntrophic interactions in a terephthalate-degrading methanogenic consortium. ISME J. 5, 122–130. 10.1038/ismej.2010.125 20686509 PMC3105669

[B70] MacIntyre S. Alldredge A. L. Gotschalk C. C. (1995). Accumulation of marine snow at density discontinuities in the water column. Limnol. Oceanogr. 40, 449–468. 10.4319/lo.1995.40.3.0449

[B71] McInerney M. J. Bryant M. P. (1981). Anaerobic degradation of lactate by syntrophic associations of *Methanosarcina barkeri* and *Desulfovibrio* species and effect of H_2_ on acetate degradation. Appl. Environ. Microbiol. 41, 346–354. 16345708 10.1128/aem.41.2.346-354.1981 PMC243697

[B72] Megonigal J. P. Hines M. E. Visscher P. T. (2004). Anaerobic metabolism: linkages to trace gases and aerobic processes, in Biochemistry, ed Schlesinger W. H. (Oxford, UK: Elsevier-Pergamon), 317–424.

[B73] Meyer K. M. Kump L. R. (2008). Oceanic euxinia in Earth history: causes and consequences. Annu. Rev. Earth Planet. Sci. 36, 251–288. 10.1146/annurev.earth.36.031207.124256

[B74] Miller C. S. Baker B. J. Thomas B. C. Singer S. W. Banfield J. F. (2011). EMIRGE: reconstruction of full-length ribosomal genes from microbial community short read sequencing data. Genome Biol. 12:R44. 10.1186/gb-2011-12-5-r44 21595876 PMC3219967

[B75] Miroshnichenko M. L. Kolganova T. V. Spring S. Chernyh N. Bonch-Osmolovskaya E. A. (2010). *Caldithrix palaeochoryensis* sp. nov., a thermophilic, anaerobic, chemo-organotrophic bacterium from a geothermally heated sediment, and emended description of the genus Caldithrix. Int. J. Syst. Evol. Microbiol. 60, 2120–2123. 10.1099/ijs.0.016667-0 19854873

[B76] Miroshnichenko M. L. Kostrikina N. A. Chernyh N. A. Pimenov N. V. Tourova T. P. Antipov A. N.. (2003). *Caldithrix abyssi* gen. nov., sp. nov., a nitrate-reducing, thermophilic, anaerobic bacterium isolated from a Mid-Atlantic Ridge hydrothermal vent, represents a novel bacterial lineage. Int. J. Syst. Evol. Microbiol. 53, 323–329. 10.1099/ijs.0.02390-0 12656191

[B77] Morris B. E. L. Henneberger R. Huber H. Moissl-Eichinger C. (2013). Microbial syntrophy: interaction for the common good. FEMS Microbiol. Rev. 37, 384–406. 10.1111/1574-6976.12019 23480449

[B78] Nobu M. K. Dodsworth J. A. Murugapiran S. K. Rinke C. Gies E. A. Webster G. (2015a). Phylogeny and physiology of candidate phylum ‘Atribacteria’ (OP9/JS1) inferred from cultivation-independent genomics. ISME J. 5, 273–286. 10.1038/ismej.2015.97 26090992 PMC4737943

[B79] Nobu M. K. Narihiro T. Rinke C. Kamagata Y. Woyke T. Liu W.-T. (2015b). Microbial dark matter ecogenomics reveals complex synergistic networks in a methanogenic bioreactor. ISME J. 8, 1710–1722. 10.1038/ismej.2014.256 25615435 PMC4511927

[B80] Noguchi H. Park J. Takagi T. (2006). MetaGene: prokaryotic gene finding from environmental genome shotgun sequences. Nucleic Acids Res. 34, 5623–5630. 10.1093/nar/gkl723 17028096 PMC1636498

[B81] Northcote T. G. Hall K. J. (1983). Limnological contrasts and anomalies in 2 adjacent saline lakes. Hydrobiologia 105, 179–194. 10.1007/BF00025187

[B82] Ogato H. Goto S. Sato K. Fujibuchi W. Bono H. Kanehisa M. (2000). KEGG: kyoto encyclopedia of genes and genomes. Nucleic Acids Res. 27, 29–34. 10.1093/nar/27.1.29 9847135 PMC148090

[B83] Overmann J. (1997). Mahoney Lake: a case study of the ecological significance of phototrophic purple sulfur bacteria, in Advances in Microbial Ecology, ed Jones J. G. (New York, NY: Plenum Press), 251–288.

[B84] Overmann J. Beatty J. T. Hall K. J. (1996b). Purple sulfur bacteria control the growth of aerobic heterotrophic bacterioplankton in a meromictic salt lake. Appl. Environ. Microbiol. 62, 3251–3258. 16535399 10.1128/aem.62.9.3251-3258.1996 PMC1388937

[B85] Overmann J. Beatty J. T. Hall K. J. Pfennig N. Northcote T. G. (1991). Characterization of a dense, purple sulfur bacterial layer in a meromictic salt lake. Limnol. Oceanogr. 36, 846–859. 10.4319/lo.1991.36.5.0846

[B86] Overmann J. Beatty J. T. Krouse H. R. Hall K. J. (1996a). The sulfur cycle in the chemocline of a meromictic salt lake. Limnol. Oceanogr. 41, 147–156. 10.4319/lo.1996.41.1.0147

[B87] Pachiadaki M. G. Lykousis V. Stefanou E. G. Kormas K. A. (2010). Prokaryotic community structure and diversity in the sediments of an active submarine mud volcano (Kazan mud volcano, East Mediterranean Sea). FEMS Microbiol. Ecol. 72, 429–444. 10.1111/j.1574-6941.2010.00857.x 20370830

[B88] Pancost R. D. Crawford N. Magness S. Turner A. Jenkyns H. C. Maxwell J. R. (2004). Further evidence for the development of photic-zone euxinic conditions during Mesozoic oceanic anoxic events. J. Geol. Soc. 161, 353–364. 10.1144/0016764903-059

[B89] Pelletier E. Kreimeyer A. Bocs S. Rouy Z. Gyapay G. Chouari R.. (2008). “*Candidatus* Cloacamonas Acidaminovorans”: genome sequence reconstruction provides a first glimpse of a new bacterial division. Appl. Environ. Microbiol. 190, 2572–2579. 10.1128/jb.01248-07 18245282 PMC2293186

[B90] Peng Y. Leung H. C. M. Yiu S. M. Chin F. Y. L. (2012). IDBA-UD: a *de novo* assembler for single-cell and metagenomic sequencing data with highly uneven depth. Bioinformatics 28, R69. 10.1093/bioinformatics/bts174 22495754

[B91] Perry K. A. Pedersen T. F. (1993). Sulphur speciation and pyrite formation in meromictic ex-fjords. Geochim. Cosmochim. Acta 57, 4405–4418. 10.1016/0016-7037(93)90491-E

[B92] Poser A. Lohmayer R. Vogt C. Knoeller K. Planer-Friedrich B. Sorokin D.. (2013). Disproportionation of elemental sulfur by haloalkaliphilic bacteria from soda lakes. Extremophiles 17, 1003–1012. 10.1007/s00792-013-0582-0 24030483

[B93] Pruesse E. Quast C. Knittel K. Fuchs B. M. Ludwig W. Peplies J.. (2007). SILVA: a comprehensive online resource for quality checked and aligned ribosomal RNA sequence data compatible with ARB. Nucleic Acids Res. 35, 7188–7196. 10.1093/nar/gkm864 17947321 PMC2175337

[B94] Rabus R. Hansen T. Widdel F. (2000). The dissimilatory sulfate and sulfur reducing bacteria, in The Prokaryotes: An Evolving Electronic Resource for the Microbiological Community, 3rd Edn., ed Dworkin M. (New York, NY: Springer Verlag), 1–87.

[B95] Ragsdale A. W. Pierce E. (2008). Acetogenesis and the Wood-Ljungdahl pathway of CO_2_ fixation. Biochim. Biophys. Acta 1784, 1873–1898. 10.1016/j.bbapap.2008.08.012 18801467 PMC2646786

[B96] R Development Core Team (2008). R: A Language and Environment for Statistical Computing. Vienna: R Foundation for Statistical Computing Available online at: http://www.R-project.org

[B97] Reinhard C. T. Planavsky N. J. Robbins L. J. Partin C. A. Gill B. C. Lalonde S. V.. (2013). Proterozoic ocean redox and biogeochemical stasis. Proc. Natl. Acad. Sci. U.S.A. 110, 5357–5362. 10.1073/pnas.1208622110 23515332 PMC3619314

[B98] Reis M. A. M. Almeida J. S. Lemos P. C. Carrondo M. J. T. (1992). Effect of hydrogen sulfide on growth of sulfate reducing bacteria. Biotechnol. Bioeng. 40, 593–600. 10.1002/bit.260400506 18601155

[B99] Rho M. Tang H. Ye Y. (2010). Fraggenescan: predicting genes in short and error-prone reads. Nucleic Acids Res. 38:e191. 10.1093/nar/gkq747 20805240 PMC2978382

[B100] Richter M. Rosselló-Móra R. Glöckner F. O. Peplies J. (2015). JSpeciesWS: a web server for prokaryotic species circumscription based on pairwise genome comparison. Bioinformatics 32, 929–931. 10.1093/bioinformatics/btv681 PMC5939971 26576653

[B101] Rinke C. Schwientek P. Sczyrba A. Ivanova N. N. Anderson I. J. Cheng J. F.. (2013). Insights into the phylogeny and coding potential of microbial dark matter Nature 499, 431–437. 10.1038/nature12352 23851394

[B102] Santos A. A. Venceslau S. S. Grein F. Leavitt W. D. Dahl C. Johnston D. T.. (2015). A protein trisulfide couples dissimilatory sulfate reduction to energy conservation. Science 350, 1541–1545. 10.1126/science.aad3558 26680199

[B103] Sato T. Atomi H. Imanaka T. (2007). Archaeal type III RuBisCOs function in a pathway for AMP metabolism. Science 315, 1003–1006. 10.1126/science.1135999 17303759

[B104] Schauder R. Preub A. Jetten M. Fuchs G. (1989). Oxidative and reductive acetyl CoA/carbon monoxide dehydrogenase pathway in *Desulfostrain autotrophicum*. Arch. Microbiol. 151, 84–89. 10.1007/BF00444674

[B105] Schink B. (1997). Energetics of syntrophic cooperation in methanogenic degradation. Microbiol. Mol. Biol. Rev. 61, 262–280. 9184013 10.1128/mmbr.61.2.262-280.1997 PMC232610

[B106] Schloss P. D. Westcott S. L. Ryabin T. Hall J. R. Hartmann M. Hollister E. B.. (2009). Introducing mothur: open-source, platform-independent, community-supported software for describing and comparing microbial communities. Appl. Environ. Microbiol. 75, 7537–7541. 10.1128/AEM.01541-09 19801464 PMC2786419

[B107] Shah N. Tang H. Doak T. G. Ye Y. (2010). Comparing bacterial communities inferred from 16S rRNA gene sequencing and shotgun metagenomics. Pac. Symp. Biocomp. 165–176. 10.1142/9789814335058_0018 21121044

[B108] Slobodkin A. I. Reysenbach A.-L. Slobodkin G. B. Kolagnova T. V. Kostrikina N. A. Bonch-Osmolovskaya E. A. (2013). *Dissulfuribacter thermophilus* gen. nov., sp. nov., a thermophilic, autotrophic, sulfur-disproportionating, deeply branching deltaproteobacterium from a deep-sea hydrothermal vent. Int. J. Syst. Evol. Microbiol. 63, 1967–1971. 10.1099/ijs.0.046938-0 23024145

[B109] Sorek R. Zhu Y. Creevey C. J. Francino M. P. Bork P. Rubin E. M. (2007). Genome-wide experimental determination of barriers to horizontal gene transfer. Science 318, 1449–1452. 10.1126/science.1147112 17947550

[B110] Sorokin D. Y. Kuenen J. G. Muyzer G. (2011). The microbial sulfur cycle at extremely haloalkaline conditions of Soda Lakes. Front. Microbiol. 2:44. 10.3389/fmicb.2011.00044 21747784 PMC3128939

[B111] Sorokin D. Y. Tourova T. P. Mußmann M. Muyzer G. (2008). *Dethiobacter alkaliphilus* gen. nov. sp. nov., and Desulfurivibrio alkaliphilus gen. nov. sp. nov.: two novel representatives of reductive sulfur cycle from soda lakes. Extremophiles 12, 431–439. 10.1007/s00792-008-0148-8 18317684

[B112] Sorokin I. I. (2002). The Black Sea: Ecology and Oceanography. Leiden: Backhuys Publishers.

[B113] Stolyar S. Van Dien S. Hillesland K. L. Pinel N. Lie T. J. Leigh J. A.. (2007). Metabolic modeling of a mutualistic microbial community. Mol. Syst. Biol. 3, 92. 10.1038/msb4100131 17353934 PMC1847946

[B114] Suen G. Weimer P. J. Stevenson D. M. Aylward F. O. Boyum J. Deneke J.. (2011). The complete genome sequence of *Fibrobacter succinogenes* S85 reveals a cellulolytic and metabolic specialist. PLoS ONE 6:e18814. 10.1371/journal.pone.0018814 21526192 PMC3079729

[B115] Swofford D. L. (2001). Paup: Phylogenetic Analysis Using Parsimony (and Other Methods) 4.0b10 Edn. Sunderland, MA: Sinauer Associate.

[B116] Tabita F. R. Hanson T. E. Li H. Statgopan S. Singh J. Chan S. (2007). Function, structure, and evolution of the RubisCO-like proteins and their RubisCO homologs. Microbiol. Mol. Biol. Rev. 71, 576–599. 10.1128/MMBR.00015-07 18063718 PMC2168653

[B117] Tamura K. Stecher G. Peterson D. Filipski A. Kumar S. (2013). MEGA6: Molecular evolutionary genetics analysis version 6.0. Mol. Biol. Evol. 30, 2725–2729. 10.1093/molbev/mst197 24132122 PMC3840312

[B118] Tatusov R. L. Natale D. A. Garkavtsev I. V. Tatusova T. A. Shankavaram U. T. Rao B. S.. (2001). The COG database: new developments in phylogenetic classification of proteins from complete genomes. Nucleic Acids Res. 29, 22–28. 10.1093/nar/29.1.22 11125040 PMC29819

[B119] Vagle S. Hume J. McLuaghlin F. MacIsaac E. Shortreed K. (2010). A methane bubble curtain in meromictic Sakinaw Lake, British Columbia. Limnol. Oceanogr. 55, 1313–1326. 10.4319/lo.2010.55.3.1313

[B120] Vetriani C. Jannasch H. W. MacGregor B. J. Stahl D. A. Reysenbach A.-L. (1999). Population structure and phylogenetic characterization of marine benthic Archaea in deep-sea sediments. Appl. Environ. Microbiol. 65, 4375–4384. 10508063 10.1128/aem.65.10.4375-4384.1999 PMC91581

[B121] Vignais P. M. Billoud B. Meyer J. (2001). Classification and phylogeny of hydrogenases. FEMS Microbiol. Rev. 25, 455–501. 10.1111/j.1574-6976.2001.tb00587.x 11524134

[B122] Voorhies A. A. Biddanda B. A. Kendall T. Jain S. Marcus D. N. Nold S. C. (2012). Cyanobacterial life at low O_2_: community genomics and function reveal metabolic versatility and extremely low diversity in a Great Lakes sinkhole mat. Geobiology 10, 250–267. 10.1111/j.1472-4669.2012.00322.x 22404795

[B123] Wang Z. Wu M. (2013). A phylum-level bacterial phylogenetic marker database. Mol. Biol. Evol. 30, 1258–1262. 10.1093/molbev/mst059 23519313

[B124] Weimer P. J. (1993). Effects of dilution rate and pH on the ruminal cellulolytic bacterium Fibrobacter succinogenes S85 in cellulose-fed continuous culture. Arch. Microbiol. 160, 288–294. 10.1007/BF00292079 8239881

[B125] Whitaker R. Banfield J. (2006). Population genomics in natural microbial communities. Trends Ecol. Evol. 21, 508–516. 10.1016/j.tree.2006.07.001 16859806

[B126] White J. R. Nagarajan N. Pop M. (2009). Statistical methods for detecting differentially abundant features in clinical metagenomic samples. PLoS Comput. Biol. 5:e1000352. 10.1371/journal.pcbi.1000352 19360128 PMC2661018

[B127] Widdel F. (1987). New types of acetate-oxidizing, sulfate-reducing *Desulfobacter* species, *D. hydrogenophilus* sp. nov., *D. latus* sp. nov., *and D. curvatus* sp. nov. Arch. Microbiol. 148, 286–291. 10.1007/BF00456706

[B128] Wrighton K. C. Thomas B. C. Sharon I. Miller C. S. Castelle C. J. VerBerkmoes N. C.. (2012). Fermentation, hydrogen, and sulfur metabolism in multiple uncultivated bacterial phyla. Science 337, 1661–1665. 10.1126/science.1224041 23019650

[B129] Wu J. H. Wu F. Y. Chuang H. P. Chen W. Y. Huang H. J. Chen S. H.. (2013). Community and proteomic analysis of methanogenic consortia degrading terephthalate. Appl. Environ. Microbiol. 79, 105–112. 10.1128/AEM.02327-12 23064332 PMC3536119

[B130] Wu M. Eisen J. A. (2008). A simple, fast, and accurate method of phylogenomic inference. Genome Biol. 9:R151. 10.1186/gb-2008-9-10-r151 18851752 PMC2760878

[B131] Ye Q. Roh Y. Carroll S. L. Blair B. Zhou J. Zhang C. L.. (2004). Alkaline anaerobic respiration: isolation and characterization of a novel alkaliphilic and metal-reducing bacterium. Appl. Environ. Microbiol. 70, 5595–5602. 10.1128/AEM.70.9.5595-5602.2004 15345448 PMC520920

[B132] Yilmaz P. Yarza P. Rapp J. Z. Glöckner F. O. (2015). Expanding the world of marine bacterial and archaeal clades. Front. Microbiol. 6:1524. 10.3389/fmicb.2015.01524 26779174 PMC4705458

[B133] Zerbino D. R. Birney E. (2008). Velvet: algorithms for *de novo* short read assembly using de Bruijn graphs. Genome Res. 18, 821–829. 10.1101/gr.074492.107 18349386 PMC2336801

[B134] Zhou X. Chen C. Wang A. Liu L. H. Ho K. L. Ren N. (2011). Rapid acclimation of methanogenic granular sludge into denitrifying sulfide removal granules. Bioresour. Technol. 102, 5244–5447. 10.1016/j.biortech.2011.01.049 21334880

