# Supplementary material for: Carbon and Sulfur Cycling below the Chemocline in a Meromictic Lake and the Identification of a Novel Taxonomic Lineage in the FCB Superphylum, Candidatus Aegiribacteria
Source: Front Microbiol. 2016 Apr 27;7:598. doi: 10.3389/fmicb.2016.00598 (PMC4846661; doi:10.3389/fmicb.2016.00598)
Supplement: Supplementary file 1 [file DataSheet1.PDF]

Table S1. Metagenome summary statistics.

	**8 m**	**Sediment**
Total Data	32 Gbp	48 Gbp
Base Pairs of Assembly	496 Mbp	781 Mbp
Total Contigs	364727	593250
% GC	44.9	48.5
N50 (bp)	2607	2583
N25 (bp)	11643	10019
Longest scaffold (bp)	446686	340441

Table S2. Marker genes used to evaluate genome completion of genome bins. Marker gene abundance is given for all genes except tRNA synthetase genes which are noted as present (Y) or absent (N).

	Genomic Bins				
	8 m			Sediments	
	**ML8_F1**	**ML8_F2**	**ML8_D**	**MLS_C**	**MLS_D**
**Marker Genes**					
RecA	1	1	1	1	1
DNA gyrase A	1	1	1	1	1
DNA gyrase B	1	1	1	1	1
RpoB	1	1	1	1	1
SecY	1	1	0	1	1
DNA RNA polymerase, beta subunit	1	1	1	1	1
elongation factor P	1	1	1	1	0
ribonuclease P	1	1	1	1	1
**Ribsosomal Proteins**					
L1	1	1	1	1	1
L2	1	1	1	1	1
L3	1	1	1	1	1
L4	1	1	1	1	1
L5	1	1	1	1	1
L6	2	1	1	1	1
L7/L12	2	1	1	1	1
L10	1	1	1	1	1
L11	1	1	2	1	1
L13	1	1	1	1	1
L14	1	1	1	1	1
L15	1	1	1	1	1
L16	1	1	1	1	1
L17	1	1	1	1	1
L18	1	1	1	1	1
L19	1	1	1	1	1
L20	1	1	1	1	1
L21	1	1	1	1	1
**Ribsosomal Proteins**					

L22	1	1	1	1	1
L23	1	1	1	1	1
L24	1	1	1	1	1
L25	1	1	2	1	1
L27	1	1	1	1	1
L28	1	0	1	1	1
L29	1	1	1	1	1
S1	1	1	1	1	1
S2	1	1	1	1	1
S3	1	1	1	1	1
S4	1	1	1	1	1
S5	1	1	1	1	1
S6	0	1	1	1	1
S7	1	1	1	1	1
S8	1	1	1	1	1
S9	1	1	1	1	1
S10	1	1	1	1	1
S11	1	1	1	1	1
S12	1	1	1	1	1
S13	1	1	1	1	1
S14	1	0	1	1	1
S15	1	1	1	1	1
S16	1	1	1	1	1
S17	1	1	1	1	1
S18	1	0	1	1	1
S19	1	1	1	1	1
S20	1	1	0	0	1
**rRNA**					
5S rRNA	1	1	0	1	1
16S rRNA	0	1	1	1	1
23S rRNA	0	0	2	1	1

**tRNA synthetase**					
ala	Y	Y	Y	Y	N

arg	Y	Y	Y	Y	Y
asp	Y	Y	Y	Y	Y
asn	Y	Y	Y	Y	Y
cys	Y	Y	Y	Y	Y
gln	Y	Y	Y	Y	Y
glu	Y	Y	Y	Y	Y
gly	Y	Y	Y	Y	Y
his	Y	Y	Y	Y	Y
ile	Y	Y	Y	Y	Y
leu	Y	Y	Y	Y	Y
lys	N	Y	Y	Y	Y
met	Y	Y	Y	Y	Y
phe	Y	Y	Y	Y	Y
pro	Y	Y	Y	Y	Y
ser	Y	Y	Y	Y	Y
thr	Y	Y	Y	Y	Y
trp	Y	Y	Y	Y	Y
tyr	Y	Y	Y	Y	Y
val	Y	Y	Y	Y	Y

Table S3. Single-copy phylogenetic marker genes used for taxonomic assignment of the genome bins.

**Ribosomal Protein**
LSU ribosomal protein L15p (L27Ae)
LSU ribosomal protein L30p (L7e)
SSU ribosomal protein S5p (S2e)
LSU ribosomal protein L18p (L5e)
LSU ribosomal protein L6p (L9e)
SSU ribosomal protein S8p (S15Ae)
LSU ribosomal protein L5p (L11e)
LSU ribosomal protein L24p (L26e)
LSU ribosomal protein L14p (L23e)
SSU ribosomal protein S17p (S11e)
LSU ribosomal protein L29p (L35e)
LSU ribosomal protein L16p (L10e)
SSU ribosomal protein S3p (S3e)
LSU ribosomal protein L22p (L17e)
LSU ribosomal protein L2p (L8e)
LSU ribosomal protein L23p (L23Ae)
LSU ribosomal protein L4p (L1e)
LSU ribosomal protein L3p (L3e)
SSU ribosomal protein S10p (S20e)

Table S4. KEGG Orthology numbers for the marker genes used for analysis of carbon, nitrogen, and sulfur cycling pathways as defined in Lauro et al., 2011; Llorens-Marès et al., 2015. Normalized marker gene ratios were calculated as described in Lauro et al., 2011; Llorens-Marès et al., 2015.

Carbon Cycle

**KEGG Orthology number**	**Gene name**	**Marker gene**
**Anaerobic carbon fixation**		
K00174	*korA, oorA*	2-oxoglutarate ferredoxin oxidoreductase subunit alpha
K00175	*korB, oorB*	2-oxoglutarate ferredoxin oxidoreductase subunit beta
K00244	*frdA*	fumarate reductase flavoprotein subunit
K01648	*aclY*	ATP citrate lyase
K00194	*cdhD, acsD*	CO dehydrogenase subunit delta
K00197	*cdhE, ascC*	CO dehydrogenase subunit gamma
**Aerobic carbon fixation**		
K00855	*PRK, prkB*	phosphoribulokinase
K01602	*rbcS*	ribulose-bisphosphate carboxylase small chain
**Fermentation**		
K00016	*LDH, ldh*	L-lactate dehydrogenase
K00169	*porA*	pyruvate ferredoxin oxidoreductase alpha subunit
K00170	*porB*	pyruvate ferredoxin oxidoreductase beta subunit
**Aerobic respiration**		
K02256	*coxI*	cytochrome c oxidase subunit I
K02262	*coxIII*	cytochrome c oxidase subunit III
K02274	*coxA*	cytochrome c oxidase subunit I
K02276	*coxC*	cytochrome c oxidase subunit III
**Methanogenesis**		
K00400		methyl coenzyme M reductase system, component A2
K00401	*mcrB*	methyl-coenzyme M reductase beta subunit
**Aerobic oxidation of methane**		
K08684		methane monooxygenase
**CO oxidation**		
K03518	*coxS*	carbon-monoxide dehydrogenase small subunit
K03519	*coxM*	carbon-monoxide dehydrogenase medium subunit
K03520	*coxL*	carbon-monoxide dehydrogenase large subunit

Nitrogen Cycle		
**KEGG Orthology number**	**Gene name**	**Marker gene**
**Nitrogen assimilation**		
K00360	*nasB*	assimilatory nitrate reductase
K00367	*narB*	assimilatory nitrate reductase
K01915	*glnA*	glutamine synthetase
K00265	*gltB*	glutamate synthase (NADPH/NADH) large chain
K00284	*gltS*	glutamate synthase (ferredoxin-dependent)
**Denitrification**		
K02305	*norC*	nitric oxide reductase subunit C
K04561	*norB*	nitric oxide reductase subunit B
K00376	*nosZ*	nitrous-oxide reductase
**Nitrogen fixation**		
K00531	*anfG*	nitrogenase delta subunit
K02586	*nifD*	nitrogenase molybdenum-iron protein alpha chain
K02588	*nifH*	nitrogenase iron protein NifH
K02591	*nifK*	nitrogenase molybdenum-iron protein beta chain
**Ammonification**		
K05904	*nrfA*	cytochrome c nitrite reductase
K03385	*nrfA*	formate-dependent nitrite reductase periplasmic cytochrome c552
**Mineralization**		
K00260	*gudB, rocG*	glutamate dehydrogenase
K00261	*gdhA, glud1*	glutamate dehydrogenase
K00262	*gdhA*	glutamate dehydrogenase
**Annamox**		
K10535	*hao/hzo*	hydroxylamine dehydrogenase/hydrazine oxidoreductase
**Nitrification**		
K10944	*amoA*	ammonia monoxygenase subunit A
K10945	*amoB*	ammonia monoxygenase subunit B
K10946	*amoC*	ammonia monoxygenase subunit C

Sulfur Cycle		
**KEGG Orthology number**	**Gene name**	**Marker gene**

**Assimilatory sulfate reduction**		
K00860	*cysC*	adenylylsulfate kinase
K00956	*cysN*	sulfate adenylyltransferase subunit 1
K00957	*cysD*	sulfate adenylyltransferase subunit 2
**Mineralization**		
K00456		cysteine dioxygenase
K01011	*sseA*	thiosulfate/3-mercaptopyruvate sulfurtransferase
**Dissimilatory sulfate reduction**		
K00394	*aprA*	adenylylsulfate reductase, subunit A
K00395	*aprB*	adenylylsulfate reductase, subunit B
K00396	*dsrA*	sulfite reductase, alpha subunit
**Sulfide oxidation**		
K00394	*aprA*	adenylylsulfate reductase, subunit A
K00395	*aprB*	adenylylsulfate reductase, subunit B
K00396	*dsrA*	sulfite reductase, alpha subunit
**Polysulfide reduction**		
K08352	*psrA*	polysulfide reductase chain A

Table S5. Top BlastP hits for single copy marker genes in the MLS_C genome. The closest affiliated sequence identified using BlastP is indication. %ID, % sequence identity between the query and hit.

	Contig	Closest affiliated sequence	% ID	Class
**Marker Genes**				
RecA	0	*Nitrospina gracilis*	70	Nitrospinia
DNA gyrase A	0	*Desulfotomaculum reducens*	55	Clostridia
DNA gyrase B	0	*Caldalkalibacillus thermarum*	62	Bacilli
RpoB	3	*Oribacteriu*m sp. FC2011	40	Clostridia
SecY	0	Dandidate Division TA06 bacterium DG_24	60	Dandidate Division TA06
DNA RNA polymerase, beta subunit	0	Candidate Division TA06 bacterium DG_24	54	Deltaproteobacteria
elongation factor P	0	uncultured delta proteobacterium Rifle_16ft_4_minimus_39832	53	Uncultured
ribonuclease P	3	*Halanaerobium praevalens*	52	Clostridia
**Ribsosomal Proteins**				
L1	0	*Thermodesulfobacterium geofontis*	57	Thermodesulfobacteria
L2	0	*Melioribacter roseus*	63	Ignavibacteria
L3	0	uncultured actinobacterium Rifle_16ft_4_minimus_550	56	Uncultured
L4	0	*Desulfosporosinus* sp. Tol-M	41	Clostridia
L5	0	*Geobacter soli*	61	Deltaproteobacteria
L6	0	Candidatus Jettenia caeni	40	Planctomycetes
L7/L12	0	Candidate Division Zixibacteria bacterium SM23_81	30	Candidate Division Zixibacteria
L10	0	*Thermaerobacter marianensis*	41	Clostridia
L11	0	Candidate Division TA06 bacterium DG_24	70	Candidate Division TA06
L13	0	*Calditerricola satsumensis*	63	Bacilli
L14	0	*Helicobacter* sp. MIT 11-5569]	73	Epsilonproteobacteria
L15	0	*Orenia marismortui*	54	Clostridia
L16	0	*Caldithrix abyssi*	72	Caldithrix
L17	0	*Paenibacillus* sp. DMB20	60	Bacilli
L18	0	*Lactobacillus saerimneri*	55	Bacilli
L19	15	Candidate Division Zixibacteria bacterium SM23_81	61	Candidate Division Zixibacteria
L20	4	*Parvularcula bermudensis*	61	Alphaproteobacteria
L21	6	*Desulfovibrio salexigens*	65	Deltaproteobacteria

**Ribsosomal Proteins**				
L22	0	*Paraoerskovia marina*	51	Actinobacteria
L23	0	Candidate Division Zixibacteria bacterium SM23_73_3	64	Candidate Division Zixibacteria
L24	0	*Porphyromonas gulae*	57	Bacteroidetes
L25	9	*Singulisphaera acidiphila*	39	Planctomycetes
L27	0	*Rubrobacter radiotolerans*	68	Actinobacteria
L28	6	Uncultured bacterium UASB14	59	
L29	0	*Thalassobacillus* sp. C254	54	Bacilli
S1	3	Candidate Division Zixibacteria bacterium SM23_81	61	Candidate Division Zixibacteria
S2	0	*Thermaerobacter marianensis*	58	Clostridia
S3	0	*Caldithrix abyssi*	58	Caldithrix
S4	3	*Geitlerinema* sp. PCC 7105	50	Cyanobacteria
S5	0	*Desulfarculus* sp. SPR	56	Deltaproteobacteria
S6	1	*Dehalococcoidia bacterium* SM23_28_1	35	Chloroflexi
S7	0	*Thermopetrobacter* sp. TC1	60	Alphaproteobacteria
S8	0	uncultured bacterium Rifle_16ft_4_minimus_4190	62	Under
S9	0	*Rhodothermus marinus*	64	Bacteroidetes
S10	0	*Syntrophothermus lipocalidus*	63	Clostridia
S11	0	*Desulfuromonas* sp. SDB	57	Deltaproteobacteria
S12	0	*Caldithrix abyssi*	83	Caldithrix
S13	0	Candidate Division Zixibacteria Zixibacteria bacterium Rifle_16ft_4_minimus_38126	71	Candidate Division Zixibacteria
S14	0	Candidatus Cloacimonas sp. SDB	67	Candidatus Cloacimonas
S15	0	Candidatus Cloacimonas sp. SDB	63	Candidatus Cloacimonas
S16	15	*Thermacetogenium phaeum*	55	Clostridia
S17	0	Glycomyces arizonensis	62	Actinobacteria
S18	1	Candidate Division Zixibacteria bacterium SM1_73	57	Candidate Division Zixibacteria
S19	0	*Gemmatirosa kalamazoonesis*	64	Gemmatimonadetes
S20	-	not present in the MLS_C genome	-	-

Figure S1. Taxonomic affiliation of all assembled metagenomic sequences at the kingdom level. Sequences affiliated with each kingdom were normalized to the total sequences in each metagenome.

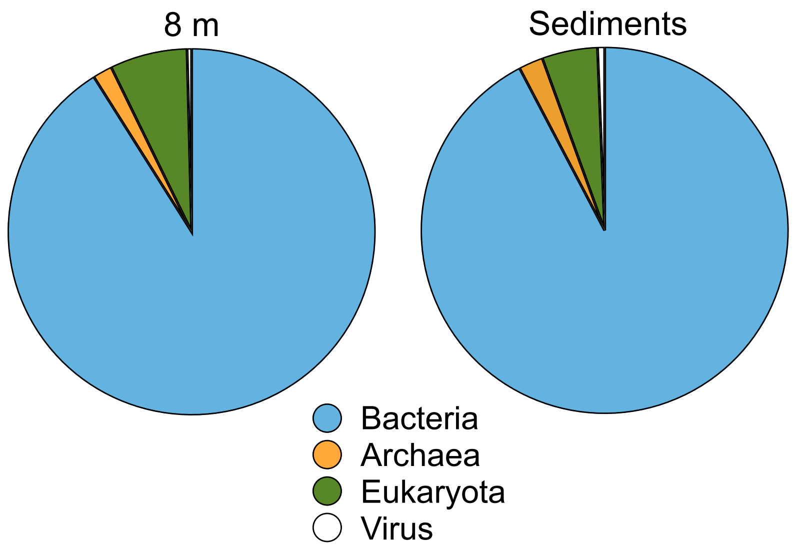

Figure S2. Phylogenetic diversity of RuBisCO below the chemocline in Mahoney Lake. Maximum likelihood phylogenetic trees constructed from CbbL and CbbM sequences mined from the 8 m and sediment metagenomes and the NCBI and IMG/M database.

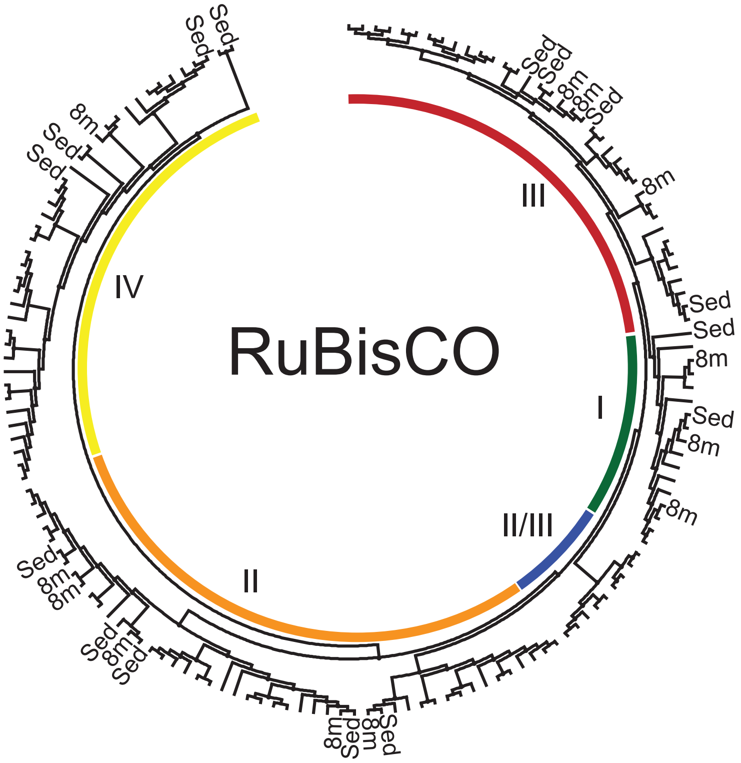

Figure S3. Maximum likelihood phylogenetic tree of 18 concatenated single-copy ribosomal proteins (Table S3) showing the taxonomic placement of the Deltaproteobacteria spp. genome bins. Bootstrap support values based on 1000 bootstrap samplings >90 are noted.

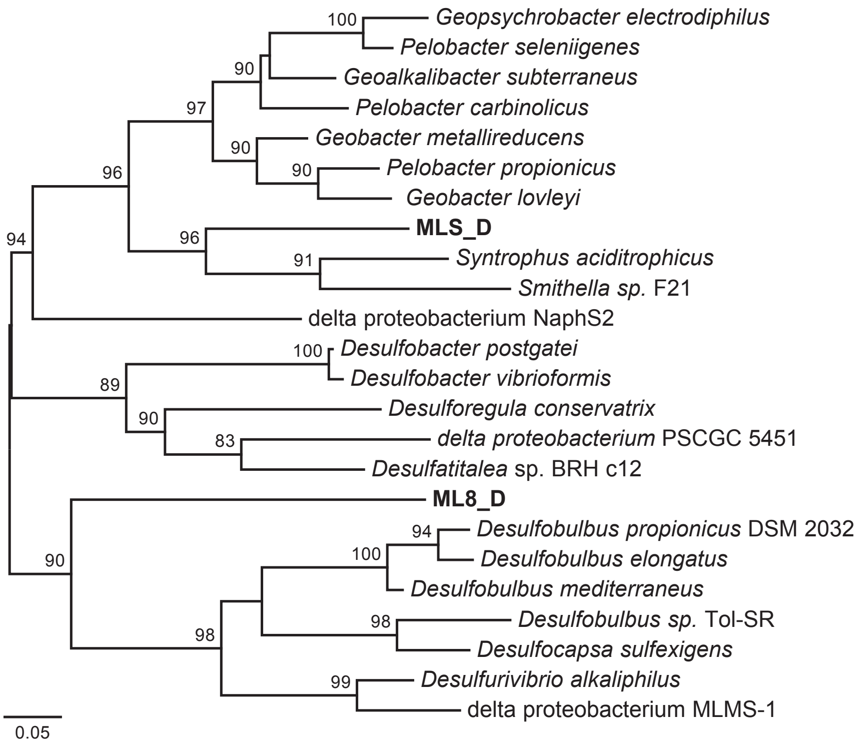

Figure S4. Maximum likelihood phylogenetic tree rpS3 proteins showing the taxonomic placement of the MLS_C genome bin. Bootstrap support values based on 1000 bootstrap samplings >85 are noted. NCBI accession numbers or IMG gene ids are shown in parentheses for references. Average nucleotide identities (ANIs) between the MLS_C genome bin and complete and partial genomes of a subset of the organisms are in parentheses in bold.

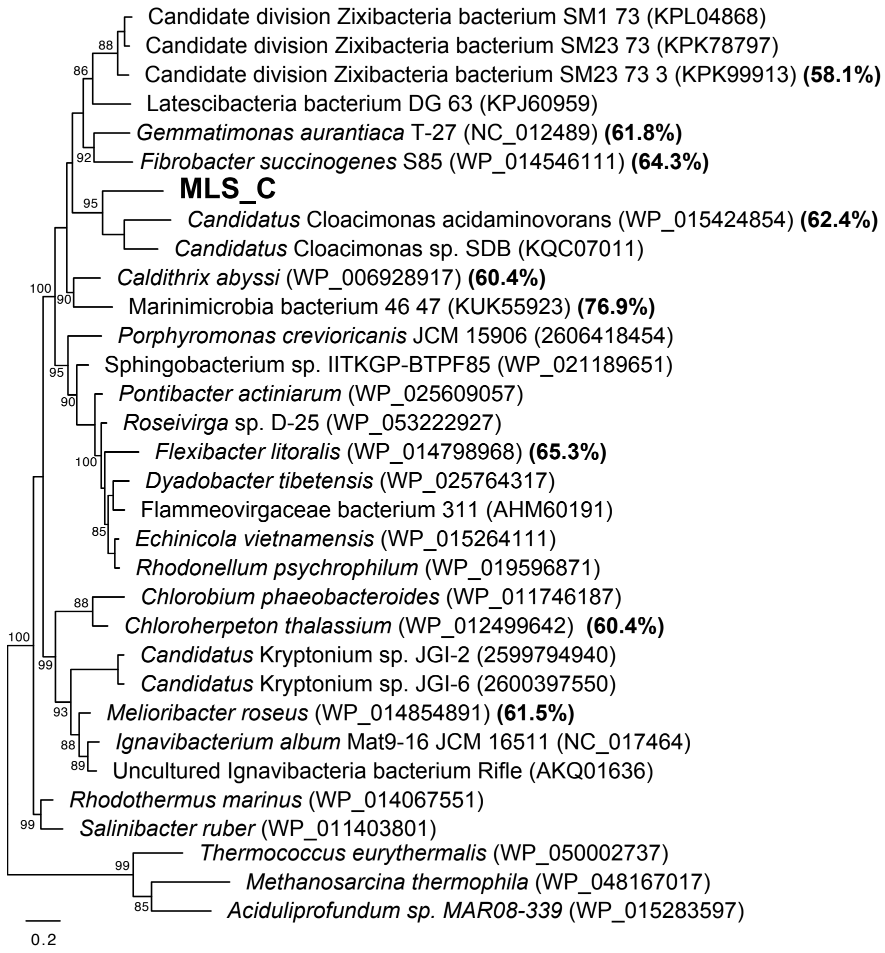

Figure S5. Hierarchical clustering of all COGs (A) and the 50 most abundant COGs (B) in each assemblage at 7 m, 8 m and in the sediments of Mahoney Lake. The abundance of individual reads matching a particular COG were converted to a fraction representing the relative contribution of each COG count to the total number of sequences assigned to COGs for each dataset (8 m and sediment) to account for different levels of sampling across multiple datasets. Values within each category are normalized across samples (see Materials and methods). Clustering analyses is based on the normalized abundance profiles of COGs.

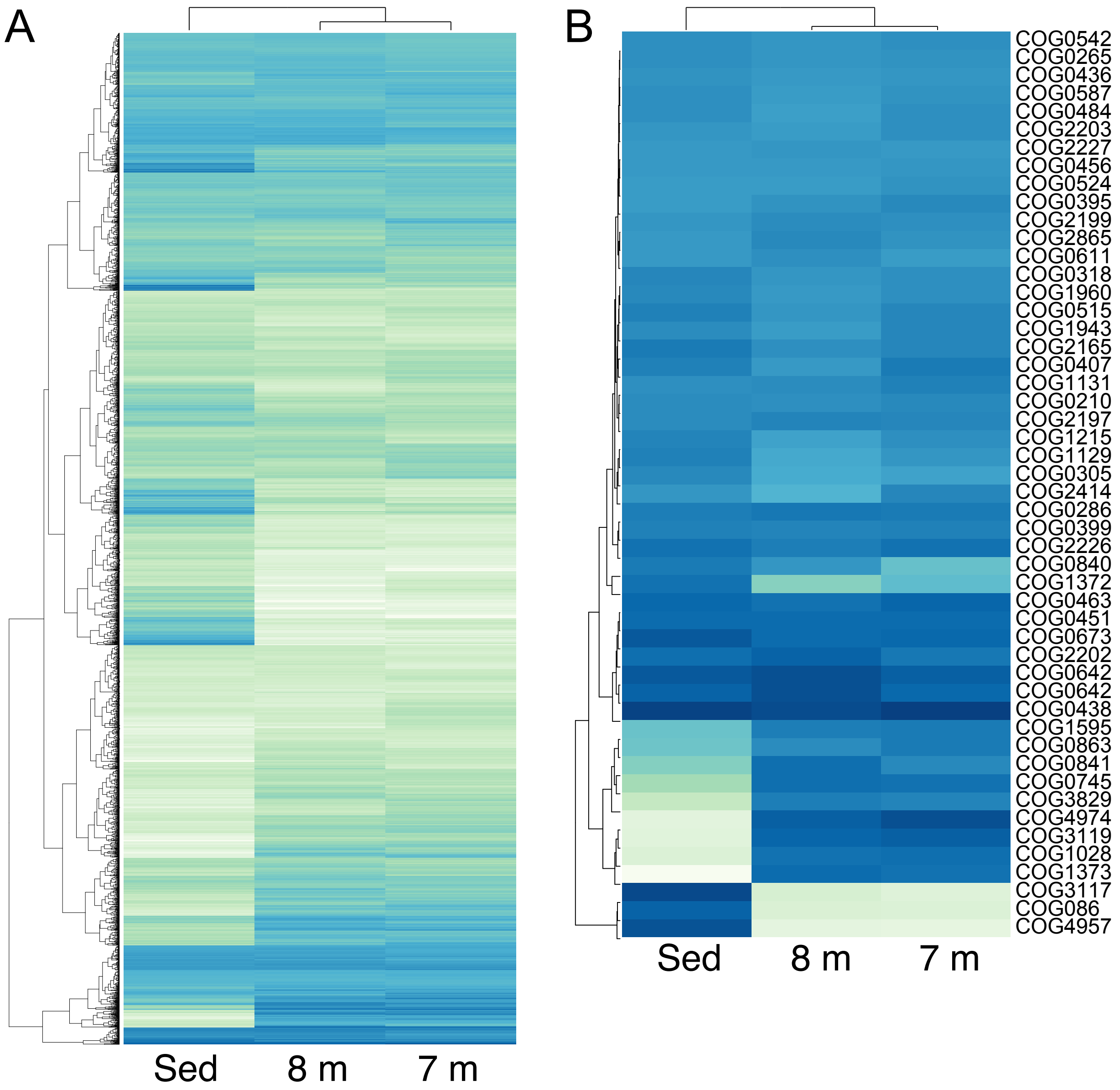

REFERENCES

Lauro, F.M., DeMaere, M.Z., Yau, S., Brown, M.V., Ng, C., Wilkins, D., Raftery, M.J., Gibson, J.A.E., Andrews-Pfannkoch, C., Lewis, M., Hofman, J.M., Thomas, T., Cavicchioli, R. (2011). An integrative study of a meromictic lake ecosystem in Antarctica. *ISME J.* 5: 879–895. doi:10.1038/ismej.2010.185

Llorens-Marès, T., Yooseph, S., Goll, J., Hoffman, J., Vila-Costa, M., Borrego, C.M., Dupont, C.L., Casamayor, E.O. (2015). Connecting biodiversity and potential functional role in modern euxinic environments by microbial metagenomics. *ISME J.* 9, 1648-1661. doi:10.1038/ismej.2014.254
